# Human Herpesviruses, Bacteria, and Fungi in Gingivitis and Periodontitis Pediatric Subjects: A Systematic Review

**DOI:** 10.3390/children12010039

**Published:** 2024-12-29

**Authors:** Federica Di Spirito, Massimo Pisano, Mario Caggiano, Giuseppina De Benedetto, Maria Pia Di Palo, Gianluigi Franci, Massimo Amato

**Affiliations:** Department of Medicine, Surgery and Dentistry, University of Salerno, Via S. Allende, 84081 Baronissi, SA, Italy; pisano.studio@virgilio.it (M.P.); macaggiano@unisa.it (M.C.); giusydb15@gmail.com (G.D.B.); mariapia140497@gmail.com (M.P.D.P.); mamato@unisa.it (M.A.)

**Keywords:** Herpesviridae, gingivitis, periodontitis, aggressive periodontitis, mouth, bacteria, virus, fungi, child

## Abstract

**Objectives:** This systematic review assesses and compares the presence and relative abundance of periodontal pathogens, human herpesviruses (HHVs), and fungi in subgingival and/or saliva samples from pediatric subjects (≤18 years of age) with periodontally healthy status and with gingivitis and/or periodontitis. **Methods:** The study protocol was conducted under the PRISMA statement and registered on PROSPERO (CRD42024593007). Data from seven studies were descriptively analyzed and qualitatively assessed through the ROBINS-1 and JBI tools. **Results:** Pediatric subjects with clinically healthy periodontium exhibited a balanced microbiome, with early colonizers (*Streptococcus* species) supporting biofilm development and late colonizers like *Fusobacterium nucleatum*, *Treponema denticola* (82.35%), and *Porphyromonas gingivalis* (29.7%) present at low levels, suggesting subclinical dysbiosis. Viruses such as HSV-I (100%), CMV (17.8%), and EBV-I (22.09%) coexisted in a likely latent state, maintained by effective immune responses. In pediatric periodontitis, biofilms were more diverse and pathogenic, with increased prevalence of *A. actinomycetemcomitans* (56.09%), *P. gingivalis* (55.4%), and *T. forsythia* (35.9%). Generalized periodontitis showed higher CMV (36.36%) and EBV-I (36.24%) prevalence than gingivitis (HSV-I 18.75%). Coinfections were frequent in periodontitis, suggesting bacterial–viral synergy in exacerbating inflammation and tissue destruction. Fungi, although not studied, may also contribute under specific conditions. **Conclusions:** These findings highlight the role of microbial interactions in periodontal health and disease progression.

## 1. Introduction

Throughout the years, several classification systems have been employed to describe periodontal diseases in children, reflecting the evolving knowledge and challenges in periodontal diseases in pediatric subjects.

The American Academy of Periodontology (AAP) first recognized “juvenile periodontitis” and “chronic marginal periodontitis” as categories of periodontal disease in 1977 [1], and in 1986 identified three subcategories of juvenile periodontitis: prepuberal, localized juvenile periodontitis, and generalized juvenile periodontitis [1]. In 1989, a new AAP classification introduced the concept of “early-onset periodontitis”, which included prepubertal periodontitis (localized or generalized), juvenile periodontitis (localized or generalized), and rapidly progressive periodontitis [2]. The key distinguishing features were the age of onset, the pattern of tooth involvement, and the progression [2]. Specifically, the localized form of juvenile periodontitis was limited to incisors and first molars into the mid to late 20s in localized forms, whereas the generalized forms were extended to multiple teeth by the age of 20s to 30s [2].

However, in 1999, a new classification was proposed by the International Workshop for Periodontal Disease and Conditions and approved by the AAP, considering the 1989 classifications’ shortcomings, as the age of onset emphasis had led to some concerns when periodontal subjects aged into a new category [3]. Indeed, the problem of age dependence emerged in instances in which the subject by age range did not fit the classifications provided in 1989 but exhibited the clinical characteristics of the type of periodontal condition [3]. In particular, when a subject had the characteristic incisor-first molar pattern of juvenile localized periodontitis but the age did not meet it, it created problems regarding classification and, therefore, diagnosis [3]. Thus, in the 1999 International Workshop for Periodontal Disease and Conditions, the age-dependent definitions or those that required knowledge of progression rates were discarded, so for these reasons, “early onset periodontitis” was replaced with “aggressive periodontitis”, categorized as the previous classification, into the localized and generalized forms [1].

The AAP and the European Federation of Periodontology (EFP) 2017 proposed a new classification of periodontal diseases and conditions [4]. One of the main changes was the unification of chronic and aggressive periodontitis under the term “periodontitis” [4] to overcome the overlap and redundancy that often occurred between previous diagnostic categories, making it challenging to apply the classification system for clinical purposes [5]. The 2017 World Workshop defines periodontitis as a microbially-associated disease that affects the supporting tissue of the tooth and leads to the loss of alveolar bone, the periodontal ligament up to the loss of the tooth itself [6].

The etiology of periodontitis recognizes the interaction between the polymicrobial flora and the immune-inflammatory response of a susceptible host, thus resulting in a complex interplay between microorganisms, host, and environmental factors [7,8].

The bacterial species involved as early and late colonizers in biofilm formation and maturation and biofilm-related gingivitis and periodontitis were divided into complexes based on a color-coded system [9].

Bacteria belonging to the red complex, which include *Porphyromonas gingivalis*, *Tannerella forsythia*, and *Treponema denticola*, and the orange complex species, such as *Prevotella intermedia*, *Fusobacterium nucleatum*, *Campylobacter rectus,* were strongly associated with periodontitis sites [9]. Recent studies have further emphasized that some bacteria species, such as *Tannerella forsythia*, are more abundant in diseased sites, showing distinct clusters of microbial communities that correlate with clinical periodontal parameters [10].

However, in pediatric subjects’ periodontitis, the role of an outlier of bacterial complexes, *Aggregatibacter actinomycetemcomitans*, a facultative Gram-negative bacterium, is considered one of the main pathogenic bacteria [11]. Its role is related to the expression of virulence factors that interact with host cells, inducing inflammatory response of the periodontal tissues [12]: the leucotoxic activity exerted by the expression of leukotoxin that determines neutrophil apoptosis, activation of macrophages in the release of interleukin-1β, and hemolytic activity [13]; the lipopolysaccharide A in the outer leaflet of the outer membrane of the bacterium involved in the induction of inflammatory host response [12]. Indeed, subjects with periodontitis and positive for *Aggregatibacter actinomycetemcomitans* showed greater bone loss than negative ones [14].

Some evidence reports the presence at periodontal sites of periodontal pathogenic bacteria and human herpesviruses (HHVs), suggesting that their interaction may result in a direct or indirect effect on the host immune response and, thus, increase disease progression, because of the lower ability of the cell infected by HHV to exert their defense against bacterial changes [15]. Periodontitis of pediatric subjects and young adults, characterized by poor biofilm accumulation, despite the rapid disease progression and destructive feature [16,17], led to the hypothesis that along with the well-established role of periodontal pathogenic bacteria, HHVs may also contribute through an interplay to the mechanisms leading to the avoidance of host defenses and disease [17]. In addition, latent HHVs might be activated due to external factors, drug therapies, stressors, or hormonal imbalances during puberty. This reactivation, often triggered by synergistic interactions with pathogen bacteria, can induce immune-inflammatory responses in the host that might potentially trigger newly onset periodontitis or reactivate the disease.

Moreover, in the mechanism of host immune defense evasion, fungi may also play a role as components of the oral microbiota [18], as suggested by some evidence that has found an association between *Candida albicans* and periodontitis [19].

However, while HHVs, periodontal pathogens, and fungi have been widely investigated in adult subjects’ periodontitis, available data on the pediatric population (≤18 years of age) are heterogeneous and low in evidence.

Although periodontitis is less common in children and adolescents than gingivitis, when it occurs, it tends to be characterized by a more localized form, often affecting the incisor-first molar pattern, as opposed to the generalized forms more commonly seen in adults [20]. The prevalence of generalized periodontitis or the molar–incisor pattern is lower in younger populations compared to adults, but early onset remains a concern due to its potential for rapid progression [20]. Differences in bacterial colonization, immune response, and the developmental stage of periodontal tissues in pediatric individuals may contribute to these variations; thus, early detection and intervention are essential to prevent long-term complications [20].

Based on these considerations, this systematic review aimed to assess and compare the presence and relative abundance of periodontal pathogens, human herpesviruses, and fungi in subgingival and/or saliva samples from pediatric subjects (≤18 years of age) with periodontally healthy status and those diagnosed with gingivitis and/or periodontitis.

## 2. Materials and Methods

### 2.1. Study Protocol

This study protocol adhered to the Preferred Reporting Items for Systematic Reviews and Meta-analyses (PRISMA) statement [21] and registered on the International Prospective Register of Systematic Review PROSPERO register (registration number: CRD42024593007), defined prior to the literature search, data extraction, and analysis.

This research focused on the addressed question “Are HHV, virus and fungi more associated with gingivitis and periodontitis in healthy pediatric subjects (≤18 years of age)?”.

The question formulation, search strategies, and study selection criteria were developed using the PICO model [22], as follows:

P—Population: Pediatric subjects (≤18 years of age) with gingivitis/periodontitis (localized or generalized aggressive periodontitis; localized or generalized prepubertal periodontitis; localized or generalized juvenile periodontitis; molar–incisor pattern periodontitis; early-onset periodontitis; gingivitis) diagnosed through current or previous accepted classifications by the American Academy of Periodontology (AAP) and the European Federation of Periodontology (EFP) [4].

I—Microbiological sampling of HHV, fungi, and periodontal pathogens in subgingival and/or saliva testing and microbiological analysis (any).

C—Comparison:

- Periodontally healthy pediatric subjects vs. pediatric subjects with gingivitis/periodontitis;
- Periodontally healthy vs. periodontitis/gingivitis sites in pediatric subjects with gingivitis and/or periodontitis.
  O—Outcome(s):
  - Primary outcome(s):
  - Presence and content of HHV, periodontal pathogens, and fungi in subgingival and/or saliva samples in pediatric subjects (≤18 years of age) with gingivitis and/or periodontitis.
  - Periodontal clinical, radiographic, and crevicular parameters.
  - Secondary outcome(s):
  - Association between periodontal pathogens bacteria, HHV, and fungi content in supra- and/or subgingival and/or saliva testing and (any) microbiological analysis.

### 2.2. Search Strategy

An electronic search was performed for records in English language on Scopus, MEDLINE/Pubmed, Web of Science, and Cochrane Library, until 16 July 2024, with no restrictions applied to date and publication status and using the following keywords and combined with the Boolean operators:

(“human herpes virus” OR HHV OR Herpesviridae OR herpes* OR cytomegalovirus OR CMV OR HCMV OR “Epstein–Barr virus” OR EBV OR “herpes simplex virus” OR HSV OR “simplex virus” OR “herpes virus 4” OR “herpes virus 6” OR “herpes virus 7” OR “herpes virus 8”) AND (periodontitis OR “juvenile periodontitis” OR “aggressive periodontitis” OR “localized aggressive periodontitis” OR “generalized aggressive periodontitis” OR “prepubertal periodontitis” OR “localized prepubertal periodontitis” OR “generalized prepubertal periodontitis” OR “molar-incisor pattern periodontitis” OR gingivitis).

AND (children OR adolescent OR infant OR juvenile OR young).

### 2.3. Study Selection and Eligibility Criteria

After the eligibility criteria definition process, two independent reviewers (G.D.B. and M.P.D.P.) independently performed the study selection, and in case of any disagreement, it was resolved through discussion by including a third reviewer (F.D.S.) if necessary.

Titles and abstracts from the electronic search were examined to remove duplicates or records not relevant to the topic. For unclear titles and abstracts, the full text was obtained before any possible exclusion.

In cases where full text was not available, contact was made with the authors.

A manual search of the references of the included articles was carried out to identify further records that were eligible for inclusion.

References of included studies were exported and managed through Mendeley Reference Manager software version 2.120.0.

Inclusion criteria were as follows: data from case reports, case series, cross-sectional, case–control, prospective, and retrospective studies in the English language without date restrictions or limitations for sample size or gender, concerning gingivitis/periodontitis in pediatric subjects (≤18 years of age) but otherwise healthy (no systemic diseases) and assessing using any of the stated methods of supra- and/or subgingival and/or saliva testing and microbiological analysis (any) samples. Participants were required to have at least one periodontitis/gingivitis site with an assessment of clinical, radiographic, and crevicular periodontal parameters and microbiological samples of supra- and/or subgingival and/or saliva testing. Gingivitis/periodontitis (periodontal conditions) diagnosis had to adhere to current or previous accepted classifications by AAP and EFP [4].

Exclusion criteria were as follows: in vitro studies or preclinical in vivo studies, systematic and narrative reviews, conference papers, book/chapters, oral communications; studies not written in the English language; adult participants subjects (>18 years of age); studies not assessing both microbiological contents of supra- and/or subgingival and/or saliva testing samples and clinical, radiographic, and crevicular parameters in pediatric subjects (≤18 years of age); pediatric subjects (≤18 years of age) with systemic diseases or syndromes (any); subjects with gingivitis/periodontitis not diagnosed through the previous or current accepted classification by AAP and EFP [4].

### 2.4. Data Extraction and Collection

Data were extracted using a standardized extraction form, developed before the search completion, by two authors independently (M.P. and G.D.B.).

The following data meeting eligibility criteria were collected from each study included in this systematic review:

Study characteristics: first author, year, journal, study design, quality assessment, funding.

Population characteristics of gingivitis/periodontitis group: participants number, mean age, gender ratio, country of origin and/or ethnicity of participants, comorbidities, anamnesis for infectious diseases, therapy for infectious diseases.

Periodontal characteristics of intervention group: periodontal conditions diagnosis, periodontal parameters (clinical, radiographic, and crevicular).

Microbiological analysis characteristics of intervention group: number of samples, type of sample, sampling methods, microbiological identification technique, sampled site, and target.

Population characteristics of clinically healthy periodontium group: participants number, mean age, gender ratio, country of origin and/or ethnicity of participants, comorbidities, anamnesis for infectious diseases, therapy for infectious diseases.

Periodontal characteristics of the control group: periodontal conditions diagnosis, periodontal parameters (clinical, radiographic, and crevicular).

Microbiological analysis characteristics of the control group: number of samples, type of sample, sampling methods, microbiological identification technique, sampled site, and target.

Outcome(s):

Viruses: HHV species detected, other viruses detected, positivity and count in PCR analysis.

Periodontal pathogen bacteria: red complex, orange complex, yellow complex, green complex, blue complex, outlier bacterial species detected, positivity and count in PCR and/or culture analysis, total anaerobic bacteria count, and/or total bacteria count.

Fungi: type and species of fungi detected, positivity, and count in PCR and/or culture analysis.

### 2.5. Data Synthesis

Data from the studies included were descriptively synthesized using Microsoft Excel Software 2019 (Microsoft Corporation, Redmond, WA, USA).

This was conducted to achieve the following aims:

To assess the clinical, radiographic, and crevicular periodontal parameters in pediatric (≤18 years of age) periodontally healthy subjects and those with gingivitis/periodontitis.

To characterize the microbiological content (HHV, periodontal pathogens, and fungi) found in HHV, periodontal pathogens, and fungi subgingival samples in pediatric (≤18 years of age) periodontally healthy subjects and those with gingivitis/periodontitis.

To characterize the microbiological content (HHV, periodontal pathogens, and fungi) found in saliva in pediatric (≤18 years of age) periodontally healthy subjects and those with gingivitis/periodontitis.

To evaluate the clinical, radiographic, and crevicular periodontal parameters in relation to subgingival and saliva microbiological content (HHV, periodontal pathogens, and fungi) in pediatric (≤18 years of age) periodontally healthy subjects and those with gingivitis/periodontitis.

To evaluate HHV and fungi content in relation to periodontal pathogens’ load in subgingival and saliva samples from pediatric (≤18 years of age) periodontally healthy subjects and those with gingivitis/periodontitis.

To compare the microbiological content (HHV, periodontal pathogens, and fungi) found in the supragingival, subgingival, and saliva samples of periodontally healthy pediatric subjects vs. pediatric subjects with gingivitis/periodontitis.

To compare the microbiological content (HHV, periodontal pathogens, and fungi) found in the supragingival, subgingival, and saliva samples of periodontally healthy vs. periodontitis/gingivitis sites in pediatric subjects with gingivitis and/or periodontitis.

### 2.6. Quality Assessment

The studies included in the present study were qualitatively assessed by two independent authors (G.D.B. and M.P.D.P.) The studies included in the present study will be qualitatively assessed by two independent authors, using the Risk of Bias In Nonrandomized Studies of Interventions (ROBINS-I) for the nonrandomized studies of interventions (freely available online on ROBINS-I tool | Cochrane Methods), and the Joanna Briggs Institute (JBI) for case report and case series (freely available online on: JBI Critical Appraisal Tools | JBI).

A third reviewer was consulted for discussion (M.P.).

## 3. Results

A total of 297 records were retrieved from electronic searches: 138 from MEDLINE/Pubmed, 133 from Scopus, 23 from Web of Science, and 3 from Cochrane Library. In total, 96 duplicates were removed prior to screening. The 201 titles/abstracts left were screened, and 125 were considered nonrelevant to the topic of the present study.

A total of 76 records were sought for retrieval. One record was not retrieved, and the authors were contacted requesting the article. No responses were obtained, and the record was excluded.

The remaining 75 records were assessed for eligibility and 62 were excluded because 23 were on adult subjects, 15 did not make it possible to extract data on pediatric subjects, 12 were reviews, 6 were not in the English language, 7 were on pediatric subjects with systemic diseases or syndromes, 2 were in vitro, 2 did not evaluate data on saliva/supra-sub gingival samples, 1 did not evaluate the HHV microbiological content, and 1 was not on gingivitis/periodontitis subjects.

A total of 6 records [17,23,24,25,26,27] were compliant with the eligibility criteria of the present systematic review and were included before the manual search.

A manual search of the reference list of the six studies [17,23,24,25,26,27] included was performed to retrieve other relevant articles on the topic of the present systematic review.

The manual search retrieved a total of 305 records. In total, 70 duplicates were removed, and of the 235 titles/abstracts left, 202 were considered nonrelevant to the topic. Of the 33 records assessed for eligibility, full-texts were screened and 32 were excluded because 13 were on adult subjects, 10 did not evaluate the HHV microbiological content, 3 did not evaluate the saliva/subgingival microbial content, 3 were reviews, 1 did not make it possible to extract data on pediatric subjects, and 1 was not on gingivitis/periodontitis subjects.

One record [28] was found to be compliant with the eligibility criteria and was included in the present systematic review through a manual search.

Finally, the present systematic review included seven articles [17,23,24,25,26,27,28] on the microbiological sampling of HHV, fungi, and periodontal pathogens in subgingival and/or saliva testing in pediatric subjects with gingivitis/periodontitis.

**Figure 1:**
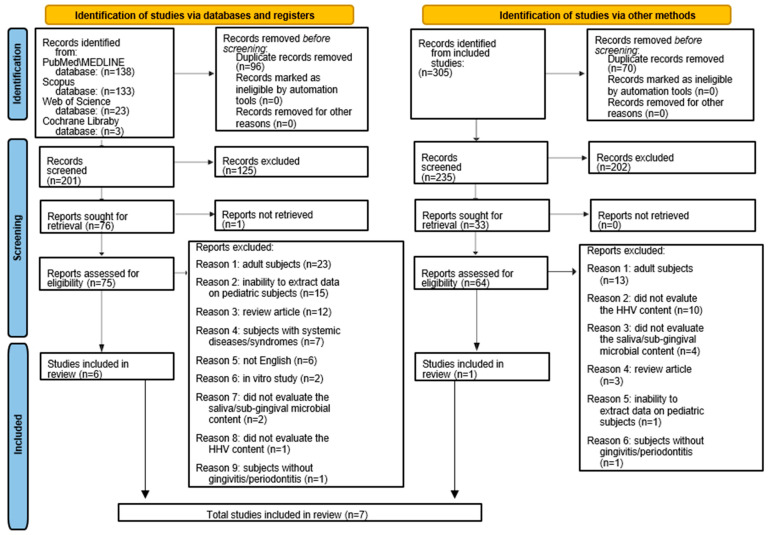
PRISMA 2020 flowchart for the study selection of systematic reviews.

Figure 1 shows the PRISMA 2020 flowchart for the study selection of electronic and manual searches.

Data from seven studies [17,23,24,25,26,27,28] concerning microbiological content (HHV, periodontal pathogens, and fungi) of saliva and/or supra–subgingival samples in pediatric subjects (≤18 years of age) with gingivitis/periodontitis were extracted and qualitatively synthesized in two tables. Table 1 reports data on the characteristics of gingivitis/periodontitis group and clinically healthy periodontium group, divided into population characteristics, periodontal parameters, and microbiological analysis characteristics. Table 2 shows the microbiological content (HHV, periodontal pathogens, and fungi) samples of gingivitis/periodontitis group and clinically healthy periodontium group.

Only data compliant with the eligibility criteria were extracted for the gingivitis/periodontitis group, so data related to other oral diseases or from nonpediatric subjects were not extracted. In the clinically healthy periodontium group, data from pediatric subjects in clinically healthy periodontal status were extracted to make a comparison, in adherence to the study selection criteria based on the PICO model.

To standardize and summarize the results, the different definitions of the periodontal conditions given by the included studies were converted to adhere to the current 2017 classification provided by the AAP and EFB [4]. Thus, studies published prior to the current classification were also standardized to be able to define the periodontal conditions of the findings examined.

The seven studies included [17,23,24,25,26,27,28] in the present systematic review were case–control.

### 3.1. Bacteria, Viruses, and Fungi in Subgingival and/or Saliva Samples from Pediatric Subjects with Periodontally Healthy Status

#### 3.1.1. Population Characteristics in Pediatric Subjects with Clinically Healthy Periodontium

In pediatric subjects with clinically healthy periodontium, the population consisted of 133 pediatric subjects [24,25,27,28].

The mean age was reported by one study [28], which was 15.6 ± 1.5 years old, and the age range was reported by one study [24], which was from 14 to 18 years old.

The gender ratio was reported by one study [28], which included 7 males and 10 females (1:1.43), while for three studies [24,25,27], gender ratio was not reported.

The country of origin was reported by two studies [24,27], which was North–Central Jamaica (n = 65) [24]; Southwestern America (n = 19) [27]. The ethnicity was reported by two studies [27,28], as follows: Pueblo-Indian (n = 19) [27]; Afro-Arab (n = 13) [28]; African (n = 4) [28].

Four studies [24,25,27,28] reported no comorbidities in 133 subjects.

Past or current infectious disease history was reported by three studies [24,25,28], in particular, it was negative in 114 subjects [24,25,28]. For 19 subjects [27], history of infectious diseases was not defined.

History of pharmacological therapy for infectious diseases was not reported by any of the study [24,25,27,28].

Periodontal condition reported was clinically healthy periodontium in 133 subjects.

#### 3.1.2. Microbiological Analysis in Pediatric Subjects with Clinically Healthy Periodontium

The total number of microbiological samples performed in pediatric subjects with clinically healthy periodontium was 417 [24,25,27,28], and in particular, the type of sampling was subgingival in 385 [24,27,28] and saliva in 32 [25].

The sampling methods reported were sterile paper points (n = 334) [24,27,28]; sterile containers (n = 32) [25].

The sampled sites reported were deepest periodontal sites (n = 82) [24,28] and periodontal sites with PPD ≤ 3.5 and BoP = 0 (n = 19) [27].

The microorganism identification techniques performed included nested PCR (n = 69) [24,27]; PCR (n = 32) [25]; culture (n = 19) [27]; LAMP method (n = 17) [28].

The targets sought by the microorganisms identification technique reported were 16S rRNA (n = 82) [24,28] and leucotoxin operon (n = 65) [24].

#### 3.1.3. Periodontal Parameters in Pediatric Subjects with Clinically Healthy Periodontium

Three studies [24,27,28] reported clinical periodontal parameters. In particular, PPD was reported by three studies [24,27,28] in 101 subjects, as follows: PPD ≤ 5 mm (n = 65) [24]; ≤3.5 mm (n = 19) [27]; 1.4 ± 0.5 mm (n = 17) [28]. CAL was reported by one study [24] in 65 subjects, which was ≤−2 mm.

BoP was evaluated by two studies [27,28] in 36 subjects, finding a BoP of 0. In 32 subjects [25], it was not possible to define clinical parameters.

In 133 subjects [24,25,27,28], it was not possible to define both radiographic and crevicular parameters.

#### 3.1.4. Bacteria in Pediatric Subjects with Clinically Healthy Periodontium

Among red complex species, *Porphyromonas gingivalis* was researched by three studies [24,27,28] in 101 pediatric subjects with clinically healthy periodontium. In particular, one study [27] identified *Porphyromonas gingivalis* through cultures, of which 1 subject resulted positive [27], whose culture counts was of 7.50 (log_10_/mL) [27], and negative in 18 subjects [27]. PCR was used by one study [24], and 22 subjects were positive and 43 negative [24]. LAMP method was used by one study [28] and 7 subjects were positive and 10 negative [28].

Of the 101 pediatric subjects with clinically healthy periodontium investigated for *Porphyromonas gingivalis* (75.93% of total clinically healthy periodontium population), a total of 30 subjects (29.70%) were positive and 71 (70.30%) negative [24,27,28].

*Treponema denticola* was investigated in one study [28] in 17 subjects (12.78% of the total clinically healthy periodontium population), of which 14 were positive (82.35%) and 3 negative (17.64%) [28].

*Tannerella forsythia* was searched by two studies [27,28] in 36 subjects (27.07% of the total clinically healthy periodontium population). In particular, one study [27] used cultures as a microorganism identification technique in which 19 subjects resulted negative [27,29], and one study [28] investigated bacterial content through LAMP, finding 9 positive subjects and 8 negative [28].

Of the 36 subjects (27.07% of the total clinically healthy periodontium population) investigated for *Tannerella forsythia* a total of 9 subjects (25.00%) resulted positive [28], and 27 (75.00%) negative [27,28].

Among orange complex bacteria, *Prevotella intermedia*/*nigriscens* was searched by one study [27] in 19 subjects (14.28% of the total clinically healthy periodontium population) through culture. In particular, 7 subjects were positive (36.84%) [27], with a count of 1.0 ± 7.50 (log_10_/mL) and 12 were negative (63.16%) [27].

*Fusobacterium nucleatum* was searched by one study [27] in 19 subjects (14.28% of the total clinically healthy periodontium population), finding 9 positive (47.37%), with a culture count of 1.4 ± 1.2 (log_10_/mL) [27] and 10 negative (52.63%) [27].

*Campylobacter rectus* was searched by one study [27] in 19 subjects (14.28% of the total clinically healthy periodontium population), through culture, finding 3 positive subjects (15.79%) and with a culture counts of 2.2 ± 1.5 (log_10_/mL) and 16 negative (84.21%) [27].

*Parvimonas micra* was sought by one study [27] through culture, in 19 subjects (14.28% of the total clinically healthy periodontium population), of which 4 resulted positive (21.05%) with a count of 3.3 ± 1.7 (log_10_/mL) [27] and 15 negative (78.95%) [27].

*Eubacterium species* were searched by one study [27] through cultures, in 19 subjects (14.28% of the total clinically healthy periodontium population), who resulted negative (100%) [27].

Green complex bacteria were searched by one study [27], in particular, investigating *Eikenella corrodens* through culture in 19 subjects (14.28% of the total clinically healthy periodontium population) [27] who resulted negative (100%).

No study has found bacteria belonging to yellow, blue, and purple complexes.

Of the bacteria outliers from the bacterial complexes, *Aggregatibacter actinomycetemcomitans* was investigated by three studies [24,27,28] in 101 subjects (75.93% of total clinically healthy periodontium population). *Aggregatibacter actinomycetemcomitans* was searched by cultures, as reported by two studies [27], in 19 subjects of which 1 subject resulted positive with a count of 0.1 (log_10_/mL) [27] and 18 were negative [27]. PCR was used by one study [24] in 65 subjects, of which 13 were positive and 52 negative [24]. One study [28] searched *Aggregatibacter actinomycetemcomitans* through LAMP method on 17 subjects, of which 1 subject resulted positive and 16 were negative [28].

Among the 101 subjects (75.93% of total clinically healthy periodontium population) investigated for *Aggregatibacter actinomycetemcomitans*, a total of 15 subjects (14.85%) resulted positive, and 86 were negative (85.15%) [24,27,28].

*Prevotella dentalis* was searched by one study [27] in culture, in 19 subjects (14.28% of the total clinically healthy periodontium population), of which all resulted to be negative (100%) [27].

*Enteric gram–rods* were evaluated by one study [27] through culture in 19 subjects (14.28% of the total clinically healthy periodontium population), which resulted negative (100%) [27].

*Capnocytophaga species* were sought by one study [27] with culture, in 19 subjects (14.28% of the total clinically healthy periodontium population) of which 6 were positive (31.58%) and with a count of 1.2 ± 1.1 (log_10_/mL) and 13 subjects were negative (68.42%) [27].

*Viridans streptococci plus* was sought by one study [27] in culture, in 19 subjects (14.28% of the total clinically healthy periodontium population), of which all were positive (100%) and with a count of 77.3 ± 12.9 (log_10_/mL) [27].

*Β-hemoltic streptococci* were investigated by one study [27] through cultures, in 19 subjects (14.28% of the total clinically healthy periodontium population), of which 1 resulted positive (5.26%) [27] and with a count of 1.1 9 (log_10_/mL), and 18 negative (94.74%) [27].

One study [27] reported the total anaerobic bacterial count, which was 7.49 ± 0.30 (log_10_/mL) [27].

No studies reported the total bacteria counts.

**Figure 2:**
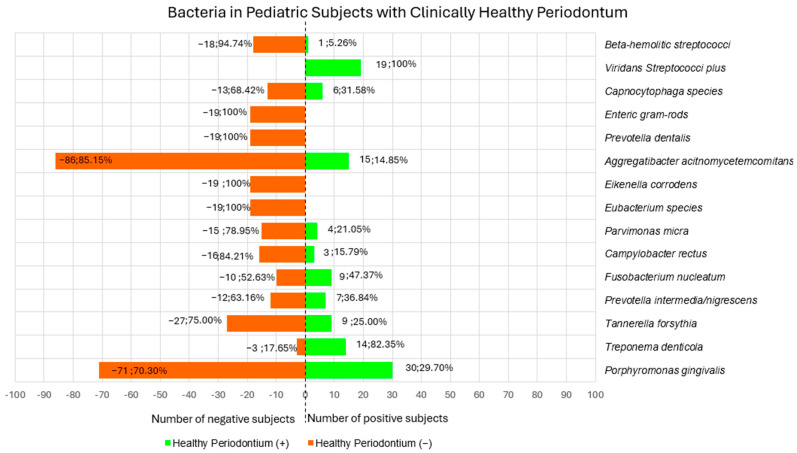
Bacteria investigated in clinically healthy periodontium pediatric subjects [24,25,27,28] sorted by number and percentage of negative (left) and positive (right) subjects. Negative data represent bacterial species that were investigated but found to be absent in the respective subjects, expressed by absolute number and percentage.

Figure 2 depicts bacteria in clinically healthy periodontium pediatric subjects.

#### 3.1.5. Viruses in Pediatric Subjects with Clinically Healthy Periodontium

HSV was investigated by one study [27] in 4 subjects (3.01% of the total clinically healthy periodontium population), who resulted negative (100%) [27].

HSV-I was investigated by one study [25] in 32 subjects (24.06% of the total clinically healthy periodontium population), who resulted positive (100%) [25].

HSV-II was investigated by one study [25] in 32 subjects (24.06% of the total clinically healthy periodontium population), of which 2 (6.25%) resulted positive and 30 (93.75%) negative [25].

CMV was investigated by four studies [24,25,27,28] in 118 subjects (88.72% of the total clinically healthy periodontium population), of which 21 resulted positive (17.80%) [24,25,28] and 97 resulted negative (82.20%) [24,25,27,28].

EBV was searched by one study [25] in 32 subjects (24.06% of the total clinically healthy periodontium population), who resulted negative (100%) [25].

EBV-I was sought by three studies [24,27,28] in 86 subjects (64.66% of the total clinically healthy periodontium population). Among them, 19 were positive (22.09%) [24,28] and negative in 67 subjects (77.91%) [24,27,28,29,30].

EBV-II was sought by one study [27] in 4 subjects (3.01% of the total clinically healthy periodontium population), who resulted negative (100%).

VZV was searched by one study [25] in 32 subjects, who resulted negative (100%).

**Figure 3:**
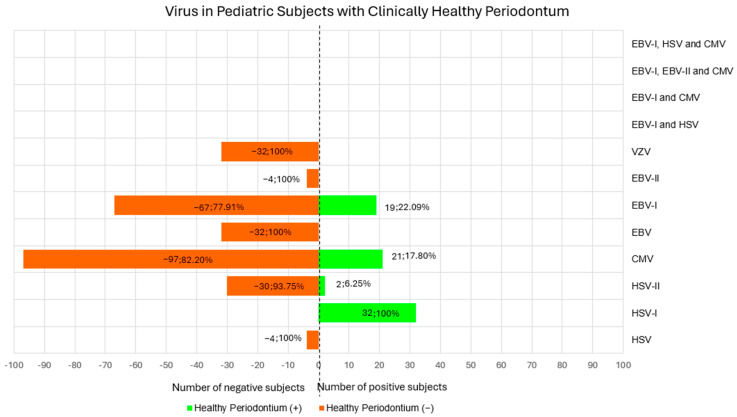
Virus investigated in clinically healthy periodontium pediatric subjects [24,25,27,28] sorted by number and percentage of negative (left) and positive (right) subjects. Negative data represent viruses that were investigated but found to be absent in the respective subjects, expressed by absolute number and percentage.

Figure 3 shows viruses in pediatric subjects with clinically healthy periodontium.

#### 3.1.6. Fungi in Pediatric Subjects with Clinically Healthy Periodontium

Fungi content was not investigated in clinically healthy periodontium pediatric subjects by any of the studies in the present systematic review.

### 3.2. Bacteria, Viruses, and Fungi in Subgingival and/or Saliva Samples from Pediatric Subjects with Gingivitis/Periodontitis

#### 3.2.1. Population Characteristic in Pediatric Subjects with Gingivitis/Periodontitis

In gingivitis/periodontitis pediatric subjects otherwise systemically healthy, the study population consisted of 102 subjects [17,23,24,25,26,27,28].

The mean age was reported by four studies [17,26,27,28], which was 15.37 ± 1.68 years old and age range from 10 [17] to 18 [24,27] years old.

The gender ratio was reported by five studies [17,23,26,27,28] which included 22 males [17,27,28] and 29 females [17,23,26,27,28] (1:1.31).

The country of origin was reported by three studies [24,26,27], which was North–Central Jamaica (n = 35) [24], Southwestern America (n = 22) [27], and Iran (n = 1) [26]. The ethnicity was reported by three studies [17,27,28], as follows: Pueblo-Indian (n = 22) [27], Afro-Arab (n = 13) [28], African–American (n = 7) [17], African (n = 4) [28], and Persian (n = 1) [17].

Past or current infectious disease history was reported by 6 studies [17,23,24,25,26,28], which in particular was negative for 80 [17,23,24,25,26,28]. For 22 subjects [27], infectious diseases history was not defined.

Periodontal conditions reported were as follows: periodontitis in 53 subjects [24,26,28]; MIPP in 33 [17,23,27]; gingivitis in 16 [25].

#### 3.2.2. Microbiological Analysis in Pediatric Subjects with Gingivitis/Periodontitis

The total number of microbiological samples performed in the gingivitis/periodontitis sites was 335 [17,23,24,25,26,27,28]; in particular, the type of sampling was 319 subgingival [17,23,24,25,26,27,28] and 16 saliva [25].

The sampling collection methods reported were sterile paper points in 319 [17,23,24,26,27,28] and sterile containers in 16 [25].

Seven studies [17,23,24,25,26,27,28] declared that no periodontal treatment was performed before microbiological sampling in the 102 subjects.

The microorganism identification techniques performed included nested PCR (n = 52) [17,24,26,27]; culture (n = 30) [17,27]; LAMP method (n = 17) [28]; PCR (n = 16) [25]; RT-PCR (n = 3) [23].

The targets sought by the microorganisms identification technique were 16S rRNA (n = 60) [17,24,28]; leucotoxin operon (n = 35) [24]; EBV DNA (n = 85) [17,24,25,26,27,28]; late MCP mRNA (n = 11) [17,23]; EBNA gene (n = 8) [17]; CMV mRNA (n = 8) [17]; CMV DNA (n = 8) [17]; IE DNA (n = 3) [23].

The sampled sites were the deepest periodontitis sites for 55 subjects [23,24,28]; periodontitis sites with PPD ≥ 6 mm in 23 [26,27]; periodontitis sites in molars and incisors in 8 [17].

#### 3.2.3. Periodontal Parameters in Pediatric Subjects with Gingivitis/Periodontitis

Seven studies [17,23,24,25,26,27,28] evaluated clinical periodontal parameters in gingivitis/periodontitis pediatric subjects, otherwise systemically healthy. In particular, PPD was reported by six studies [17,23,24,26,27,28], in 86 subjects, specifically 53 with periodontitis [24,26,28]; 33 with MIPP [17,23,27] as follows: ≥5 mm (n = 35 periodontitis) [24]; 7.2 ± 0.9 mm (n = 22 MIPP) [27]; 6.9 ± 1.5 mm (n = 17 periodontitis) [28]; from 5 to 9 mm (n = 8 MIPP) [17]; from 6 to 10 mm (n = 3 MIPP) [23]; from 6 to 9 mm (n = 1 periodontitis) [29]. Clinical parameters on bleeding were reported by four studies [25,26,27,28] in 56 subjects, specifically 22 with MIPP [27]; 18 with periodontitis [26,28] and 16 with gingivitis [25]. Parameters evaluated were BoP, which was 1 in 39 subjects (n = 22 with MIPP; n = 16 gingivitis; n = 1 periodontitis) [25,26,27]; BoP expressed as a percentage value of bleeding sites, with a value of 62.4% (n = 17 periodontitis) [28].

CAL was assessed by two studies [24,28] in 52 subjects with periodontitis, which was ≤−3 mm (n = 35) [24]; −4.8 ± 1.2 (n = 17 periodontitis) [28].

Both radiographic and crevicular parameters were not assessed by any study included in the present systematic review regarding systemically healthy pediatric subjects with gingivitis/periodontitis.

#### 3.2.4. Bacteria in Pediatric Subjects with Gingivitis/Periodontitis

Among the red complex bacteria, *Porphyromonas gingivalis* was investigated by three studies [17,24,28] in 74 subjects (72.55% of the total population); among them, 52 (70.27%) had periodontitis [24,28] and 22 (29.33%) MIPP [27]. One study [27] reported the identification of the bacteria through cultures, whose resulted positive in 14 subjects (n = 14 MIPP) [27] with a count of 5.80 ± 7.0 (log_10_/mL) and negative for 8 subjects (n = 8 MIPP) [27]. One study [28] reported the use of the LAMP identification method, which resulted positive in 14 subjects (n = 14 periodontitis) [28] and negative in 3 (n = 3 periodontitis) [28]. Nested PCR was used by one study [24], whose resulted positive in 27 subjects (n = 27 periodontitis) and negative in 8 (n = 8 periodontitis).

Of the 74 subjects investigated for *Porphyromonas gingivalis*, a total of 55 subjects (74.32%) were positive (n = 41, 55.40% periodontitis and 14, 18.92% MIPP) [24,27,28] and 19 negative (25.67%) (n = 11, 14.87% periodontitis; n = 8, 10.81% MIPP) [24,27,28].

*Treponema denticola* was investigated in one study [28] in 17 subjects (16.67% of the total population) with periodontitis, through the LAMP method, of which 12 were positive (70.59%) (n = 12, 70.59% periodontitis) [28] and 5 negative (29.41%) (n = 5, 29.41% periodontitis) [28].

Two studies [27,28] reported the investigation of *Tannerella forsythia* in 39 subjects (38.23% of the total population); specifically, 22 subjects (56.41%) had MIPP [27] and 17 (43.59%) periodontitis [28]. One study [28] identified the bacteria through LAMP method, finding 13 positive subjects (n = 13 periodontitis) [28] and 4 negative ones (n = 4 periodontitis) [28]; one study [27] used cultures as identification technique, of which 14 subjects resulted positive (n = 14 MIPP), with a count of 1.8 ± 2.3 (log_10_/mL) and 8 were negative (n = 8 MIPP) [27].

Among the 39 subjects investigated for *Tannerella forsythia*, a total of 27 subjects (69.23%) resulted positive (n = 14, 35.90% MIPP; n = 13, 33.33% periodontitis) and 12 negative (30.77%) (n = 8, 20.51% MIPP; n = 4, 10.26% periodontitis) [27,28].

Among the orange complex bacteria, one study [27] reported *Prevotella intermedia/nigriscens* identification in 22 subjects (21.57% of the total population) with MIPP [27] using culture. In particular, 19 subjects resulted positive (86.36%) (n = 19, 86.36% MIPP) [27], with a count of 4.3 ± 4.2 (log_10_/mL), and 3 resulted negative (13.64%) (n = 3, 13.64% MIPP) [27].

*Fusobacterium nucleatum* was searched by one study [27] in 22 subjects (21.57% of the total population) with MIPP through culture method [27]. In particular, 21 subjects resulted positive (95.45%) (n = 21, 95.45% MIPP), with a count of 2.6 ± 3.1 (log_10_/mL) and 1 negative (4.55%) (n = 1, 4.55% MIPP) [27].

*Campylobacter rectus* was investigated in one study [27] in 22 subjects (21.57% of the total population) with MIPP through cultures [27]. 16 subjects (72.73%) (n = 16, 72.73% MIPP) resulted positive, with a count of 4.8 ± 3.8 (log_10_/mL) and 6 subjects (27.27%) (n = 6, 27.27% MIPP) were negative [27].

*Parvimonas micra* was investigated by one study [27] in 22 subjects (21.57% of the total population) with MIPP through cultures. In particular, 16 subjects (72.73%) resulted positive [27] (n = 16, 72.73% MIIP), with a bacteria count of 7.1 ± 6.9 (log_10_/mL) and 6 (27.27%) were negative (n = 6, 27.27% MIPP) [27].

*Eubacterium species* were investigated in one study [27] in 22 subjects (21.57% of the total population) with MIPP, through culture. In particular, 3 subjects (13.64%) were positive (n = 3, 13.64% MIPP) and with a count of 3.2 ± 1.8 (log_10_/mL) [27] and negative in 19 subjects (86.36%) [27].

Among green complex bacteria reported, *Eikenella corrodens* was investigated by one study [27] in 22 MIPP subjects (21.57% of the total population) through culture. In particular, 2 subjects were positive (9.09%) (n = 2, 9.09% MIPP) with a count of 3.0 ± 4.1 (log_10_/mL) [27] and negative in 20 subjects (90.91%) (n = 20, 90.91% MIPP) [27].

No study has found bacteria belonging to the purple complex.

Of the bacteria outliers from the bacterial complexes, *Aggregatibacter actinomycetemcomitans* was investigated by four studies [17,24,27,28] in 82 subjects (80.39% of the total population) of which 52 with periodontitis (63.41%) [24,28] and 30 with MIPP (36.59%) [17,27]. One study [24] performed nested PCR for microbiological analysis technique, finding 18 positive subjects (n = 18 periodontitis) and 17 negative (n = 17 periodontitis) [24]; one study [27] examined *Aggregatibacter actinomycetemcomitans* through culture founding 10 subjects positive (n = 10 MIPP) with a count of 3.1 ± 3.3 (log_10_/mL) and 12 negative (n = 12 MIPP) [27]; one study [28] used the LAMP method finding 12 positive (n = 12 periodontitis) and 5 negative (n = 5 periodontitis) subjects [28]; one study performed both culture and PCR, with 4 positive subjects at culture (n = 4 MIPP) [17] with a count expressed as a percentage of total bacterial counts of 0.3%; 0.6%; 2.6%; and 4.2% (n = 4 MIPP) [17] and negative in 4 (n = 4 MIPP) [17] and 4 positive at PCR as well (n = 4 MIPP) and 2 subjects (n = 2 MIPP) negative at PCR (n = 2 MIPP) [17].

Of the 82 subjects (80.39% of the total population) investigated for *Aggregatibacter actinomycetemcomitans* a total of 46 subjects (56.10%) resulted positive (n = 30, 36.58% periodontitis; n = 16, 19.51% MIPP) and 36 (43.90%) (n = 22, 26.84% periodontitis; n = 14, 17.07% MIPP) [17,24,27,28].

*Enteric gram–rods* were sought by one study [27] in 22 subjects (21.57% of the total population) with MIPP, of which 1 resulted positive (4.55%) (n = 1, 4.55% MIPP) with a count of 8.3 (log_10_/mL) and 21 (95.45%) negative (n = 21, 95.45% MIPP) [27].

*Prevotella dentalis* was searched by one study [27] in 22 subjects (21.57% of the total population) with MIPP through culture, finding 3 positive subjects (13.64%) (n = 3, 13.64% MIPP) with a count of 2.5 ± 3.7 (log_10_/mL) and 19 negative subjects (86.36%) (n = 19, 86.36% MIPP) [27].

*Capnocytophaga species* were investigated by one study [27] in 22 subjects (21.57% of the total population) with MIPP through culture, finding 10 positive subjects 45.45% (n = 10, 45.45% MIPP) with a count of 3 ± 3.2 (log_10_/mL) and 12 negative subjects (54.55%) (n = 12, 54.55% MIPP) [27].

*Viridans streptococci plus* was searched by one study [27] in 22 subjects (21.57% of the total population) with MIPP through culture, finding 22 positive subjects (100%) (n = 22, 100% MIPP) with a count of 38.8 ± 11.7 (log_10_/mL) [27].

*Β-Hemolytic streptococci* were investigated by one study [27] in 22 subjects (21.57% of the total population) with MIPP, and resulted positive in 7 (31.82%) (n = 7, 31.82% MIPP), with a bacterial culture count of 3.4 ± 5.4 (log_10_/mL) and negative in 15 (68.18%) [27].

One study [27] reported the total anaerobic bacterial culture count, which was 7.97 ± 0.55 (log_10_/mL). No studies reported the total bacterial count.

**Figure 4:**
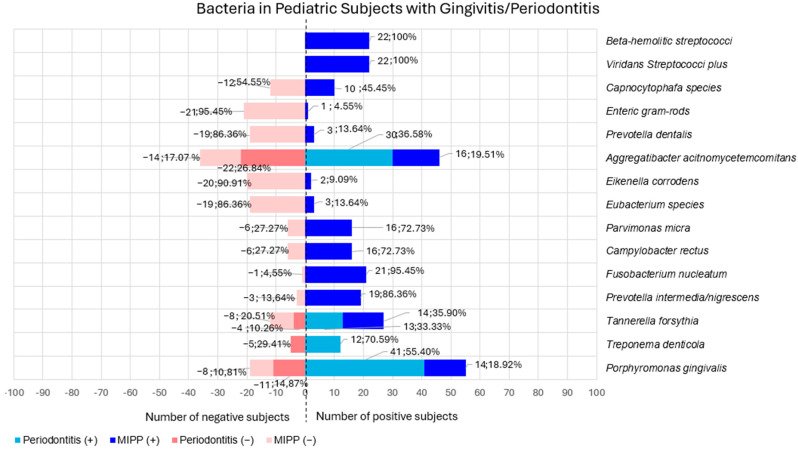
Bacteria investigated in gingivitis/periodontitis pediatric subjects [17,23,24,25,26,27,28] sorted by number and percentage of negative (left) and positive (right) subjects, and by periodontal condition (periodontitis, MIPP).

Figure 4 shows bacteria found in gingivitis/periodontitis pediatric subjects.

#### 3.2.5. Viruses in Pediatric Subjects with Gingivitis/Periodontitis

HSV was searched by three studies [17,26,27] in 17 subjects (16.67% of total population), of which 16 had MIPP (94.12%) [17,27] and 1 periodontitis (5.88%) [26]. Among the 17 subjects investigated for HSV, 4 subjects were positive (23.53%) (n = 4, 23.53% MIPP) [17] and 13 negative (76.47%) (n = 12, 70.59% MIPP; n = 1, 5.88% periodontitis) [17,26,27].

HSV-I was sought by one study [25] in 16 subjects (15.69% of the total population) with gingivitis, of which 3 resulted positive (18.75%) and 13 negative (81.25%) [25].

HSV-II was sought by one study [25] in 16 subjects (15.69% of the total population) with gingivitis, of which 3 resulted positive (18.75%) and 13 negative (81.25%) [25].

CMV was investigated by seven studies [17,23,24,25,26,27,28] in 88 subjects (86.27% of total population), specifically 53 with periodontitis (60.23%) [24,26,28], 16 with gingivitis (18.18%) [25] and 19 with MIPP (21.59%) [17,23,27]. Among them, CMV resulted positive in 44 subjects (50%) (n = 32, 36.36% periodontitis; n = 12, 13.64% MIPP) [17,23,24,26,27,28] and negative in 44 (55.84%) (n = 21, 23.86% periodontitis; n = 16, 18.19% gingivitis; n = 7, 7.95% MIPP) [17,24,25,27,28].

EBV was sought by one study [25] in 16 subjects (15.69% of the total population) with gingivitis. EBV was negative in the 16 subjects (100%) [25].

EBV-I was researched by five studies [17,24,26,27,28] in 69 subjects (67.65% of the total population), in particular, 53 had periodontitis (76.81%) [24,26,28] and 16 MIPP (23.19%) [17,27]. EBV-I was found to be positive in 32 subjects (46.38%) (n = 25, 36.24% periodontitis; n = 7, 10.14% MIPP) [17,24,27,28], negative in 37 (53.62%) (n = 28, 40.58% periodontitis, n = 9, 13.04% MIPP) [17,24,26,27,28].

EBV-II was investigated by two studies [17,27] in 16 subjects (15.69% of the total population) with MIPP, of which 1 resulted positive (6.25%) [17] and 15 negative (93.75%) [17,27].

VZV was researched by one study [25] in 16 subjects with gingivitis (15.69% of the total population), and was found to be negative in the 16 subjects investigated (100%) [25].

Among the HHV-positive cases, coinfections were evaluated by three studies [17,27,28] in 33 subjects (32.35% of the total population); among them, 17 had periodontitis (51.52%) [28] and 16 MIPP (48.48%) [17,27].

EBV-I and CMV coinfection was sought in 33 subjects (32.35% of total population); of them, 17 had periodontitis (51.52%) [28] and 16 MIPP (48.48%) [17,27]. Of the 33 subjects investigated for EBV-I and CMV coinfection, 10 subjects (30.30%) resulted positive (n = 8, 24.24% periodontitis; n = 2, 6.06% MIPP) and 23 (69.70%) negative (n = 14, 42.42% MIPP; n = 9, 27.27% periodontitis) [17,27,28].

CMV, EBV-I, and EBV-II coinfection was investigated in eight subjects (7.84% of total population) with MIPP [17]. Among them, two subjects were positive (25.00%) and six negative 75.00%) [17].

CMV, EBV-I and HSV coinfections were sought in eight subjects (7.84% of total population) with MIPP [17]. Among them, two subjects were positive (25.00%) and six negative 75.00%) [17].

CMV and HSV coinfection was investigated in eight subjects (7.84% of total population) with MIPP [17]. Among them, one subject (12.5%) resulted positive and seven (87.5%) negative [17].

EBV-I and HSV coinfection was investigated in eight subjects (7.84% of total population) with MIPP [17]. Of the eight subjects investigated, one (12.5%) resulted positive and seven (87.5%) negative [17].

**Figure 5:**
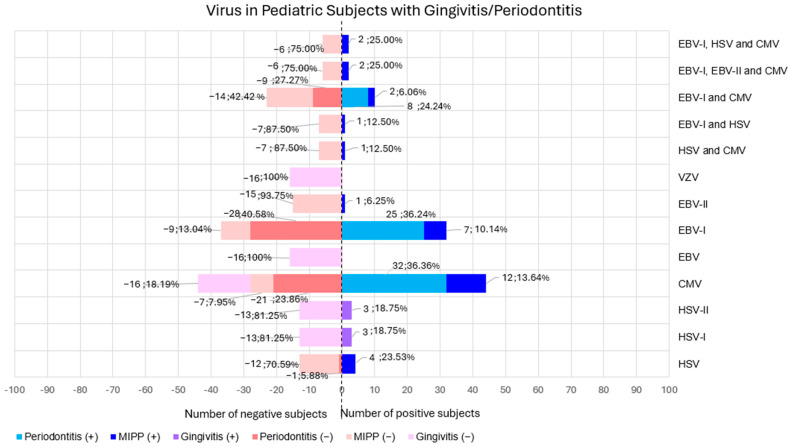
Virus investigated in gingivitis/periodontitis pediatric subjects [17,23,24,25,26,27,28] sorted by number and percentage of negative (left) and positive (right) subjects and by periodontal condition (periodontitis, MIPP, gingivitis).

Figure 5 shows viruses found in gingivitis and periodontitis pediatric subjects, but otherwise healthy.

#### 3.2.6. Fungi in Pediatric Subjects with Gingivitis/Periodontitis

Fungi content in pediatric subjects with gingivitis/periodontitis was not investigated by any of the studies included in the present systematic review.

### 3.3. Quality Assessment

As the studies included in the present systematic review were nonrandomized studies, the ROBINS-I tool was employed to assess the bias risk and the quality assessment and reported in Table S1 (Supplementary File S1).

## 4. Discussion

The present systematic review aimed to assess and compare the presence and relative abundance of periodontal pathogens, human herpesviruses (HHVs), and fungi in subgingival and/or saliva samples from pediatric subjects (≤18 years of age) with periodontally healthy status and those diagnosed with gingivitis and/or periodontitis.

The oral microbiome is a well-balanced dynamic community of microorganisms, encompassing bacteria, viruses, fungi, archaea, and protozoa, that establish a symbiotic relationship with the host through homeostatic mechanisms [31,32]. The composition of the oral microbiome is profoundly influenced by interactions between the host, which may culminate in a transition from a healthy “eubiotic” state to a diseased “dysbiotic” state, which may lead to gingivitis and periodontitis [31].

Although the oral microbiome is predominantly composed of bacterial species, other biomes such as the viral (“virobiome”) and fungal (“mycobiome”) are also relevant in discerning the shift from healthy or diseased state [32,33]. Accordingly, some pediatric subjects and young adults with periodontitis showed poor biofilm accumulation despite the rapid disease progression and destructive feature, which led to the hypothesis that, along with the role of periodontal pathogenic bacteria, HHVs may also contribute through an interplay to the mechanisms leading to the avoidance of host defenses and to the shift to the disease [34].

Indeed, the polymicrobial synergy and dysbiosis theory suggest that a synergistic polymicrobial community can drive disease through several mechanisms such as the expression of specific molecules, adhesins, ligands, proteolytic enzymes, and pro-inflammatory agents [11]. Such cooperative interspecies mechanisms, not seen in a single pathogen, enable tissue-destructive host response [11]. Periodontal bacteria only exhibit pathogenic potential when host defenses or microenvironmental conditions favor susceptibility to inflammation, maintaining eubiosis otherwise [11]. Consequently, in the development of gingivitis and periodontitis, not only bacterial biofilms but also interactions with viruses and fungi contribute to eliciting an immune-inflammatory response in a susceptible host [11,35].

### 4.1. Bacteria, Viruses, and Fungi in Subgingival and/or Saliva Samples from Pediatric Subjects with Periodontally Healthy Status

#### 4.1.1. Bacteria in Pediatric Subjects with Clinically Healthy Periodontium

Pediatric subjects with clinically healthy periodontium had PPD values ≤ 5 mm in 65 subjects, ≤3.5 mm in 19, and 1.4 ± 0.5 mm in 17, while CAL values were ≤−2 mm in 65 subjects. In such healthy periodontal conditions, oral bacterial biofilms are expected to maintain a balanced state called eubiosis through pH, oxygen, nitrate, and nisin regulation [36]. Specifically, early colonizers, like saccharolytic aerobic and facultative bacteria (mainly *Streptococcus* species), use glycoproteins and salivary mucins as nutrients to form microcolonies by secreting extracellular substances (polysaccharides, lipids, DNA) [36], aiding in bacterial adhesion, maturation, and protection [36,37]. Adhesion is further promoted by appendages like fimbriae or pili in species such as *Streptococci*, *Actinomyces*, and *Porphyromonas gingivalis* [38,39], beyond fibrils, typical of some *Streptococcus* species, *Prevotella intermedia* and *nigriscens* [40]. Late colonizers (*Fusobacterium nucleatum*, *Treponema denticola*, *Tannerella forsythia*, *Prevotella intermedia*, *Porphyromonas gingivalis*, *Aggregatibacter actinomycetemcomitans*) exploit binding sites on early colonizers to enhance biofilm maturation [40], and interact through coaggregation, forming bonds with complementary binding sites [41,42].

Biofilms overall develop layers with aerobic species on the surface and anaerobic species in deeper regions, where *Fusobacterium nucleatum* plays a key role in linking aerobic and anaerobic bacteria, promoting biofilm diversity [40,43,44]. Although biofilm accumulation in childhood and adolescence are recognized predisposing cofactor for the development of gingivitis and periodontitis [45], Plaque Index (PI) and Full Mouth Plaque Score (FMPS) records were not provided in the included studies, so it was not possible to define oral hygiene status of the pediatric subjects with clinically healthy periodontium examined. Moreover, biofilm accumulation has often been correlated with BoP+ also in pediatric subjects [45], which was absent in the 36 pediatric subjects under study. Given these considerations, it may be assumed that the pediatric subjects with clinically healthy periodontium investigated may have adequate biofilm control, probably due to their age (ranging between 14 and 18 years of age with a mean age of 15.6 ± 1.5), favoring a greater awareness of oral hygiene maintenance and biofilm control compared to younger subjects [46].

Although the crevicular, as well as the radiographic, parameters were not reported in the studies included in the present systematic review, it may be assumed that, as in young adult individuals, no imbalance between pro- and anti-inflammatory cytokine patterns occurred in the pediatric subjects, thus contributing to the clinically healthy periodontium status. Indeed, in young adults, subjects with periodontitis were associated with a higher ratio of interleukin-1 beta and interleukin-10 levels compared to clinically healthy periodontium subjects [47].

Subgingival sampling was conducted in molars and incisors by some studies, while others did not specify the sampling site. This is noteworthy because bacterial counts can vary depending on the sampling location, with higher counts often found in posterior or interdental sites compared to mid-buccal sites [48]. Therefore, the presence of a mature biofilm and pathogenic periodontal species in clinically healthy subjects may reflect these interindividual differences in biofilm accumulation as well as the variability in sampling collection sites.

However, in the subgingival and salivary samples in the 133 subjects with clinically healthy periodontium, the presence of species belonging to the late biofilm colonizers was found, including Gram-negative anaerobic bacteria such as *Porphyromonas gingivalis* (29.7%), *Treponema denticola* (82.35%), *Tannerella forsythia* (25. 00%), *Prevotella intermedia/nigriscens* (36.84%), *Fusobacterium nucleautum* (47.37%), and *Campylobacter rectus* (15.79%), and facultative Gram-negative anaerobic bacteria, including *Aggregatibacter actynomycetemcomitans* (14.85%) and *Capnocytophaga species* (31.58%), suggestive of the composition of a mature and well-organized biofilm that may lead to an increased susceptibility to the shift to disease.

It is worth noting that the oral microbiome changes its composition, maturation, and complexity through the various stages of dental development, and age, from deciduous dentition to mixed and permanent dentition [49]. In the deciduous dentition, the presence of *Pseudomonaceae*, *Enterobacteriaceae,* and *Pasturacellae* (genus *Aggregatibacter*) is prevalent, while in the transition from deciduous to permanent dentition, the presence of *Veillonellaceae* and *Prevotella* increases, the proportions of *Bacteroidetes* and *Spirochaetes* are higher with increasing age, and at puberty, due to hormonal changes and increased bioavailability of nutrients, the oral environment is enriched with anaerobic Gram-negative species [39,49]. Although the stage of dentition of pediatric subjects is not reported in the studies included in this systematic review, in relation to the average age of the participants, that found in the samplings might be considered congruent with the composition change of a biofilm of older or pubertal/prepubertal age pediatric subjects, such as the presence of Gram-negative anaerobic species and a high prevalence of *Spirocaethes* (*Treponema denticola*, 82.35% of the subjects investigated) and *Bacteriodetes* (*Porphyromonas gingivalis*, 29.70%; *Prevotella intermedia* 36.84%), and might be consistent with a clinically healthy periodontium.

The majority of pediatric subjects with clinically healthy periodontium were negative for periodontal pathogens, which may support their healthy periodontal status.

However, some subjects with an equally healthy periodontium were positive for red complex pathogens, such as *Tannerella forsythia* and *Porphyromonas gingivalis* (29.70%) (25.00%). According to the keystone hypothesis, even low levels of specific pathogens can destabilize the microbiome and initiate an inflammatory response that may set the stage for disease onset [18]. The presence of *Porphyromonas gingivalis* in these healthy subjects could suggest a transient dysbiotic state or a subclinical imbalance in the biofilm that did not progress to disease. This consideration may imply that pathogenic bacteria alone may not be sufficient to drive disease, and, similarly, their absence may not guarantee health. Increasing evidence suggests that shifts toward disease may depend on microbial abundance and ecological succession rather than the presence of individual pathogens [50,51]. This aligns with previous studies in adult subjects showing that *Porphyromonas gingivalis* can be present in health without necessarily leading to disease [31].

In pediatric subjects with clinically healthy periodontium, the orange complex bacteria found were *Prevotella intermedia/nigriscens* (36.84%), *Fusobacterium nucleatum* (47.37%), *Campylobacter rectus* (15.79%), and *Parvimonas micra* (21.05%). Orange complex bacteria were also detected, including *Prevotella intermedia/nigriscens* (36.80%), *Fusobacterium nucleatum* (47.40%), *Campylobacter rectus* (15.80%), and *Parvimonas micra* (21.10%).

Among outliers bacteria, *Aggregatibacter actinomycetemcomitans* was found in 14.9%, *Capnocytophaga* species in 31.6%, *Viridans streptococci* in 100%, and *β-hemolytic streptococci* in 5.30% of subjects. *Aggregatibacter actinomycetemcomitans* was found in 14.85% of clinically healthy pediatric subjects in the present systematic review. Although commonly linked to periodontitis in younger individuals [14,52], *Aggregatibacter actinomycetemcomitans* has multiple serotypes and clonal forms; some strains, such as the highly virulent jp2 genotype, are associated with disease development, while other non-jp2 genotypes have been found in healthy sites [52,53]. Thus, its presence in these subjects may be attributable to non-jp2 genotypes, which may be compatible with a healthy periodontium status.

On the other side, bacteria from the green or yellow complexes were rarely investigated in clinically healthy pediatric subjects, with a potential underestimation of bacteria typically associated with periodontal health [9]. Moreover, the mechanisms underlying the transition from a eubiotic to a dysbiotic state remain unclear in the development of gingivitis and periodontitis [54].

These findings are consistent with the ecological plaque hypothesis [7], which posits that potentially pathogenic species, including those within the red and orange complexes and outliers such as *Aggregatibacter actinomycetemcomitans*, are present in healthy pediatric subjects at low concentrations. Their presence may contribute to maintaining a balanced eubiotic state, clinically reflected by a healthy periodontium. This suggests that these species do not induce the ecological stress necessary to trigger disease or activate the host’s immune-inflammatory response. Such outcomes are likely influenced by a combination of local, genetic, and environmental factor [55], beyond biofilm accumulation [39], as supported by both non-plaque-specific and plaque-specific theories, including the ecological plaque hypothesis and the keystone pathogen hypothesis [7,18].

#### 4.1.2. Viruses in Pediatric Subjects with Clinically Healthy Periodontium

The human oral “virobiome” is characterized by variable and highly individualized contents of viruses that contribute actively to the maintenance of host dynamic homeostasis [31].

Many viruses identified in the oral cavity belong to prokaryotic viruses, known as bacteriophages, which, through their replication, can exhibit a lytic or lysogenic cycle, influencing bacterial composition and functions, with the potential ability to rearrange human bacterial communities [56,57]. Some bacteriophages are associated with oral commensal bacteria, and in maintaining the state of dynamic equilibrium with the host, they contribute to the elimination of bacterial species, limiting their growth and maintaining oral health status [57]. Numerous prokaryotic bacteria have been found in salivary and subgingival samples, and their role in the shift from eubiosis to dysbiosis has not yet been elucidated [56]. Accordingly, although not presently investigated, those prokaryotic viruses may also have played a role in maintaining periodontal health.

Conversely, among the Eukaryotic viruses generally found in the oral microbiome of asymptomatic healthy subjects, especially belonging to *Herpesviridae*, *Papilloma viridae,* and *Anelloviridae* families [31], several HHVs were detected in the 133 pediatric individuals with healthy periodontium., potentially because the gingival epithelium, as well as saliva, could act as reservoir sites of viruses in latent form, which may not result in the establishment of disease processes [56,58].

Indeed, HHVs possess the ability to cross host barriers with specific tissue tropism and establish lifelong infections that can persist in either an active or latent state [56]. During latency, the virus evades the host’s innate and adaptive immunity, reactivating spontaneously or under stressors that compromise immune defenses [34]. Evidence regarding their presence in healthy periodontal individuals is inconsistent, with some studies reporting a complete absence in subgingival samples and others detecting lower proportions compared to periodontitis cases [34,59]. In clinically healthy periodontium, HHVs are hypothesized to exist predominantly in a latent or nontranscriptional state [34], coexisting in a balanced homeostasis with the bacterial biofilm and other components of the oral microbiome without triggering an immune response. This hypothesis aligns with the findings of the present review, where most children with clinically healthy periodontium tested negative for HHVs, while a minority were positive. The transient presence of HHVs as bystanders cannot be excluded. However, growing evidence suggests that HHVs may play a more active role in driving the transition from health to disease, potentially influencing the microbial dynamics and immune response of the host.

Specifically, HSV-I was recorded in 32 (100% of those tested), and HSV-II in 2 (6.25%) subjects. Noteworthily, most pediatric subjects with clinically healthy periodontium had no current or past infections of HSV-1. However, since primary HHV infections commonly occur in childhood, some subjects may have experienced asymptomatic or subclinical infections, which may explain the presence of some viruses in clinically healthy periodontium pediatric subjects. For instance, HSV-I was found to be present in 100% of pediatric subjects tested. HSV-I is associated with primary infection of orofacial area during childhood, which may be manifest with herpetic gingivostomatitis or be asymptomatic; and can establish latency in the trigeminal and spinal ganglia [34]. Some evidence reported the presence of HSV in sulcular epithelium in reactivation state before the clinical manifestation of lip lesions that frequently occur in the secondary infection [58]. It may be hypothesized that the HSV-1 presence in these clinically healthy pediatric subjects may indicate a latent viral state or a preclinical phase preceding the secondary infection’s clinical orofacial manifestations. This may suggest that HSV-1 detected in healthy periodontium could represent an early indicator of potential secondary manifestations following a prior asymptomatic infection in early childhood.

It has also been suggested that other HHVs, such as EBV or CMV, may have a tropism for the gingival sulcus and they may target specifically sulcular and junctional epithelium. Accordingly, CMV and EBV-I were detected in 21 (17.80%) and 19 (22.09%) periodontally healthy pediatric subjects. Similarly, Vincent-Bugnas et al. [60] found these viruses in clinically healthy periodontium subjects, although in a lower frequency if compared to periodontitis subjects, suggesting the presence of EBV also in healthy periodontium of pools of B-cells infected by the virus in its latency stage. The results of the present systematic review may confirm the previous findings, as EBV-I resulted positive in 22.09% and CMV in 17.80% in clinically healthy periodontium pediatric subjects, so it may be likely that the disease-provoking mechanism was not triggered, as EBV may lay latent in the junctional and sulcular B-cells and CMV in macrophages or T-cells.

It may also be possible that in the case of an active infection by HHVs, such as by CMV, it may have been controlled by the humoral or cell-mediated immunity of the pediatric subject, thus justifying the clinically healthy periodontal status, since, as pointed out earlier, the mechanism involved in the disease consists of the ability to subvert the host defenses and trigger the immune-inflammatory response [48]. Indeed, it has been demonstrated in animal models, and hypothesized for humans, that natural killer cells increase as an attempt to control CMV infection [61]. One of the mechanisms exploited by CMV as an attempt to subvert the host is to prevent the recognition of natural killer cells [61]. It could be hypothesized that the presence of natural killer cells in the context of gingival fluid, along with a proper immuno-inflammatory competence of the host, may have resulted in infection control in subjects with clinically healthy periodontium, thus not resulting in disease.

#### 4.1.3. Fungi in Clinically Healthy Periodontium Pediatric Subjects

Although fungi were not analyzed in this systematic review, it is plausible that the presence of certain periodontal pathogens, such as *Aggregatibacter actinomycetemcomitans* (positive in 14.85% of clinically healthy subjects), did not trigger disease due to limited interactions with other biofilm components, such as viruses or fungi, and an effective host immune response. Yang et al. [62] investigated the supragingival biofilm in adolescents undergoing orthodontic treatment and found that *Candida albicans* was less prevalent in subjects with clinically healthy periodontium than in those with gingivitis, correlating with a lower biofilm load and complexity.

### 4.2. Bacteria, Viruses, and Fungi in Subgingival and/or Saliva Samples from Pediatric Subjects with Gingivitis and Periodontitis

#### 4.2.1. Bacteria in Pediatric Subjects with Gingivitis and/or Periodontitis

Although bacterial content was not investigated in pediatric subjects with gingivitis, in periodontitis cases, a broader biofilm spectrum and increased prevalence of pathogenic species, such as *Porphyromonas gingivalis*, *Fusobacterium nucleatum*, *Prevotella intermedia/nigriscens*, and *Aggregatibacter actinomycetemcomitans*, were observed compared to clinically healthy periodontium. While pathogenic species were also detected in healthy subjects, their abundance was significantly lower.

Periodontitis is a relatively uncommon condition in pediatric individuals compared to adults [20]. Evidence suggests that the prevalence of the diverse forms of periodontitis varies among children and adolescents from 2 to 6 percent, depending on geographic, demographic, genetic, environmental, and socioeconomic factors [63]. These prevalence data underscore the need to explore the microbial communities and the broader host–microbe interactions in younger individuals that lead to periodontal tissue destruction in generalized or molar–incisor pattern periodontitis.

For instance, *A. actinomycetemcomitans* was absent in 85.15% of periodontally healthy subjects, but only in 43.91% of periodontitis cases. Similarly, *P. gingivalis* was undetected in 70.30% of healthy subjects, compared to just 25.68% of those with periodontitis. These findings highlight the stark contrast in bacterial profiles between healthy and diseased periodontal states, underscoring the pathogenic shift associated with periodontitis [31,39].

The bacteria most strongly associated with molar–incisor pattern periodontitis (MIPP) in pediatric subjects included *Aggregatibacter actinomycetemcomitans* (19.51%), *Porphyromonas gingivalis* (18.92%), *Treponema denticola* (70.59%), *Fusobacterium nucleatum* (95.95%), and *Campylobacter rectus* (72.73%). Among these, the role of *A. actinomycetemcomitans*, particularly the Jp2 genotype serotype b, is well documented in the pathogenesis of MIPP, with greater severity observed in young individuals [14,64]. In detail, the presence of *Aggregatibacter actinomycetemcomitans*, especially in Jp 2 genotypes, is associated with a higher progression of worsening periodontal clinical parameters such as CAL or PD [52]. Indeed, in PPD higher values, a greater total bacteria count was also found, which may support the previous findings [52]. Coherently, PPD values found were 7.2 ± 0.9 mm (n = 22), from 5 to 9 mm (n = 8), and from 6 to 10 mm (n = 3). Although *Aggregatibacter* play a role in MIPP and generalized periodontitis in pediatric and young individuals, the shift to the disease depends on the host, bacteria species characteristics and features, and the abundance and variety of biofilm, as well as environmental and genetic factors, but it also may reside in an interplay between other microorganisms, such as HHVs [59,64].

Accordingly, other bacterial species also contribute to MIPP development, with their prevalence varying based on the subject’s dentition status [64]. Previous studies have reported the presence of *A. actinomycetemcomitans* alongside *Campylobacter rectus*, *Fusobacterium nucleatum*, and *Gemella morbillorum* in individuals with permanent dentition [65]. Although the dentition status of subjects could not be determined in this review, the findings align with existing evidence. Given the average age of the subjects (15.37 ± 1.68 years; range 10–18 years), the observed bacterial composition likely reflects a biofilm associated with permanent dentition.

In subjects with generalized pattern periodontitis, the highest prevalence was found in bacterial species such as *Porphyromonas gingivalis* (55.40%), *Aggregatibacter actinomycetemcomitans* (36.58%), *Tannerella forsythia* (35.90%), and *Treponema denticola* (70.59%), which were unexpectedly higher when compared to individuals with clinically healthy periodontium.

However, the pathogenic traits of these bacteria, such as *Porphyromonas gingivalis* altering local pH or producing proteases, and *Aggregatibacter actinomycetemcomitans* generating endotoxins, cannot solely explain the disease, as they were found in subjects with clinically healthy periodontium. This observation suggests that while these bacteria contribute to the disease, other microorganisms may also play a role in its onset and progression, particularly in pediatric subjects undergoing hormonal changes during puberty and immune system maturation, potentially affecting the immune-inflammatory response [12,66].

#### 4.2.2. Viruses in Pediatric Subjects with Gingivitis and Periodontitis

In pediatric subjects with gingivitis, herpes simplex virus type 1 (HSV-I) was detected in 18.75% of cases, demonstrating a higher prevalence compared to other forms of periodontal disease. This finding aligns with HSV tissue tropism for oral epithelial cells and the sulcular epithelium, where it increases epithelial permeability and promotes vascular infiltration by inflammatory cells [32]. Because HSV-I is primarily confined to superficial epithelial layers, it is more likely to manifest as gingivitis rather than periodontitis, which involves deeper connective tissue inflammation. Notably, 81.25% of gingivitis cases tested negative for HSV-I, likely due to the virus being in a latent state. Additionally, none of the pediatric gingivitis cases (0%) tested positive for varicella-zoster virus (VZV), likely because of VZV’s affinity for skin epithelial cells, where it typically manifests as a vesicular rash rather than affecting the oral mucosa [34].

Among pediatric subjects with periodontitis, cytomegalovirus (CMV) and Epstein–Barr virus type 1 (EBV-I) were the most frequently detected herpesviruses. CMV was found in 36.36% of cases with generalized periodontitis and 13.64% of cases with molar–incisor pattern periodontitis (MIPP). Similarly, EBV-I was detected in 36.24% of generalized periodontitis cases and 10.14% of MIPP cases. In contrast, CMV was absent in gingivitis cases, suggesting its preferential association with more severe forms of periodontal disease. This difference may stem from CMV’s ability to interact with toll-like receptors (TLRs), particularly TLRs 2, 7, and 9, which are more highly expressed in periodontitis lesions compared to gingivitis [34,61].

HHVs in periodontitis may shift from a latent to an active state under inflammatory stimuli, such as those induced by biofilm, hormonal changes, or stress, contributing to disease progression [16]. For instance, CMV can latently infect gingival fibroblasts [67] and reactivate under such stimuli, producing pro-inflammatory cytokines like interleukin-1β and tumor necrosis factor. These mediators promote inflammatory responses and osteoclast differentiation, ultimately leading to periodontal tissue destruction if the host immune response fails to counteract these effects [16].

EBV-I was also prevalent, with 36.24% detected in generalized periodontitis and 10.14% in MIPP cases, while EBV-II was detected in 6.25% of generalized periodontitis cases. Similar to CMV, EBV reactivation may be influenced by the presence of periodontopathogenic bacteria such as *Porphyromonas gingivalis* and *Fusobacterium nucleatum.* These bacteria can produce metabolites like butyric acid, which trigger viral activation and exacerbate the inflammatory state [68,69]. Notably, *P. gingivalis* was the most prevalent bacterium in pediatric periodontitis (55.40% in generalized periodontitis; 18.92% in MIPP), suggesting a synergistic relationship between EBV and bacterial pathogens in disease progression.

The association between HHVs and clinical periodontal parameters in pediatric subjects was also notable. Periodontal probing depths (PPDs) in periodontitis cases ranged from 5 to 9 mm, with a mean of 7.2 ± 0.9 mm in MIPP and 6.9 ± 1.5 mm in generalized periodontitis. Clinical attachment loss (CAL) in periodontitis cases reached up to −4.8 ± 1.2 mm. CMV positivity was more frequently observed in pediatric subjects with higher PPD and CAL values, suggesting a potential link between CMV infection and increased periodontal tissue destruction. This finding is consistent with prior studies in adults, where CMV was associated with more severe periodontal parameters [68].

Generalized periodontitis exhibited a higher prevalence of HHVs compared to MIPP. This may be partly due to the larger study population with generalized periodontitis, but also reflects the broader and more complex bacterial biofilm associated with this condition. Generalized periodontitis is characterized by a diverse microbial community, whereas MIPP is more commonly associated with a limited number of pathogens, such as *Aggregatibacter actinomycetemcomitans* and *P. gingivalis* [31,41,64].

Coinfections involving HHVs were more frequently detected in generalized periodontitis than in MIPP, supporting a stronger viral–bacterial interaction in the former. For example, CMV and HSV coinfections were found in 12.5% of generalized periodontitis cases, while EBV-I and CMV coinfections were observed in 24.24% of generalized cases compared to 6.06% in MIPP. These interactions likely enhance immune evasion mechanisms, promoting viral activation and further contributing to disease progression.

#### 4.2.3. Fungi in Pediatric Subjects with Gingivitis and Periodontitis

Although none of the studies in this review investigated fungi in subgingival plaque or saliva, their role in the pathogenesis of periodontitis, including in young populations, remains debated [19,70,71]. Indeed, studies have reported increased levels of *Candida albicans* and *Candida dubliniensis* in Moroccan adolescents with periodontitis compared to periodontally healthy subjects [71], even if the presence of yeast has not been strongly linked to disease severity. Interestingly, certain pathogenic bacteria, such as *Aggregatibacter actinomycetemcomitans*, which produce quorum-sensing molecules like autoinducer-2, may inhibit fungal hyphal growth [69]. Furthermore, acute inflammatory changes in the periodontal microenvironment may suppress yeast colonization and their transition to opportunistic pathogens [19]. However, it may also be hypothesized that fungi can transition from being harmless commensals to pathogenic organisms under favorable environmental conditions, working in synergy with bacteria and viruses to undermine host defenses and contribute to the development of gingivitis or periodontitis [19,70,71].

Candidal hyphae are more commonly detected in subgingival specimens from immunocompromised or diabetic individuals [19]. Given that the pediatric subjects included in this review were systemically healthy, fungi were unlikely to be present. Nevertheless, further studies are required to explore the potential role of fungi in the etiopathogenesis of gingivitis and periodontitis in healthy pediatric populations. Such research should also investigate fungi’s interactions with periodontal bacterial species and viruses, particularly HHVs, and their contribution to the transition from periodontal health to disease.

### 4.3. Strength, Limitations, and Future Perspectives

The present systematic review has several limitations that should be considered when interpreting the findings. Most included studies were conducted between 1998 and 2017, with only one study published in 2024. Advances in microbial detection techniques during this period may have resulted in variability in the accuracy of microbiological data. Additionally, the use of heterogeneous and nonstandardized sampling methods likely influenced the consistency of the results. Another limitation to acknowledge is the potential impact of the risk of bias in the included studies which may have impacted the findings. Variations in study design, sample size, population characteristics, and methodologies across the included studies could have introduced biases, which may have affected both the validity of individual studies and the overall reliability of the synthesized evidence.

Geographical differences also present a challenge; most participants in the studies that reported origins were of Afro-descendant populations, potentially limiting the generalizability of the findings to other demographic groups. The lack of standardized periodontal parameters, as well as missing data on radiographic and crevicular assessments, further constrained the comprehensive interpretation of results.

Future research should prioritize larger, geographically diverse cohorts to capture variations in microbial composition and environmental influences on disease. The underexplored role of fungi, particularly *Candida albicans*, in pediatric periodontal diseases warrants further investigation. Additionally, studies should address treatment effects on microbiological content, which were not examined in this review, to inform targeted therapeutic approaches.

Emerging technologies, such as metagenomic and metatranscriptomic analyses, offer significant potential for identifying and monitoring specific pathogens and their responses to interventions. The development of chairside detection tools for HHVs, bacteria, and fungi could further enhance the clinical management of pediatric periodontal diseases. Future research should focus on elucidating the interplay between HHVs, pathogenic bacteria, and fungi in gingivitis and periodontitis, as well as their mechanisms for evading host defenses and contributing to disease progression.

## 5. Conclusions

Pediatric subjects with clinically healthy periodontium exhibited a balanced oral microbiome. Early colonizing bacteria like *Streptococcus* play a critical role in biofilm development, while late colonizers such as *Fusobacterium nucleatum*, *Treponema denticola*, and *Porphyromonas gingivalis* contribute to biofilm complexity through interactions with early colonizers. Although pathogenic species like *P. gingivalis* (29.7%) and *T. denticola* (82.35%) were detected, their low levels suggest a transient or subclinical dysbiosis that did not progress to disease.

Among viruses, HSV-I (100%), CMV (17.8%), and EBV-I (22.09%) were detected, likely in a latent state coexisting with the bacterial biofilm. Effective immune responses, including natural killer cell activity, likely prevented disease, maintaining a balanced host-microbe relationship.

Fungi, though not directly analyzed, were likely less prevalent in clinically healthy periodontium. Overall, these findings suggest that a stable, well-regulated microbiome and competent immune response are key to maintaining periodontal health despite the presence of potentially pathogenic microbes.

While gingivitis is more prevalent in pediatric populations, presently retrieved data focused primarily on periodontitis. Pathogenic bacteria such as *Porphyromonas gingivalis*, *Fusobacterium nucleatum*, *Prevotella intermedia*, and *Aggregatibacter actinomycetemcomitans* were significantly more abundant in diseased states. *A. actinomycetemcomitans* was present in 56.09% of periodontitis cases compared to 14.85% in healthy subjects, likely due to the Jp2 genotype, which is linked to increased severity. Generalized periodontitis exhibited a broader bacterial spectrum, including *Tannerella forsythia* (35.9%) and *Treponema denticola* (70.59%), suggesting that bacterial abundance and host–environment interactions drive disease progression.

Herpesviruses were more prevalent in periodontitis than gingivitis, with CMV (36.36%) and EBV-I (36.24%) frequently detected in generalized periodontitis. HSV-I, found in 18.75% of gingivitis cases, appears confined to superficial tissues. Coinfections, such as CMV and EBV, were more common in generalized periodontitis, suggesting synergy between viruses and bacteria in exacerbating periodontal tissue destruction. CMV’s association with increased PPD and CAL underscores its role in inflammatory-mediated damage. Periodontitis in pediatric subjects has been attributed to the loss of virulence of *Aggregatibacter actinomycetemcomitans*; however, HHVs may also contribute by transitioning to a latent state under host defense pressure. HHVs may further explain the pathogenetic pattern of molar–incisor pattern periodontitis in children, given their site-specificity. Through synergistic interactions with pathogenic bacteria, HHVs contribute to inflammation, collagen degradation, and bone resorption.

Although fungi were not investigated in this review, prior studies suggest that under favorable conditions, fungi may potentially interact with bacteria and viruses to contribute to periodontal disease. Further research is required to elucidate their role.

## Figures and Tables

**Table 1:** Characteristics of pediatric subjects with clinically healthy periodontium and gingivitis/periodontitis: study (first author; year; study design; reference; quality assessment; funding); population characteristics (population number; mean age; country/ethnicity; comorbidities; anamnesis for infectious diseases; therapy for infectious diseases); periodontal conditions (periodontal conditions; periodontal parameters; clinical, radiographic, crevicular parameters); microbiological analysis (number of samples; type of sample; sampling methods; time after periodontal treatment; microbiological identification technique; sampled site; target).

Studies	Pediatric Subjects with Gingivitis/Periodontitis				Pediatric Subjects with Clinically Healthy Periodontium			
	Population	Periodontal Conditions		Microbiological Analysis	Population	Periodontal Conditions		Microbiological Analysis
Contreras A., 1998 Oral Microobiol Immunol Case-control study [23] Moderate risk No funding	Population: n.3 Mean age: range 14–17 yo n.3 Gender ratio: 3F Country/Ethnicity: MD Comorbidities: None n.3 Anamnesis for infectious disease: none n.3 Therapy for infectious disease: NA	Periodontal conditions: MIPP n.3		N. of sample: n.18 Type of sample: subgingival n.18 Sampling methods: sterile paper points n.18 Time after periodontal treatment: none n.3 Microbiological identification technique: RT-PCR n.3 Sampled site: deepest periodontitis sites n.3 Target: IE DNA, late MCP mRNA n.3				
		Periodontal parameters:						
		Crevicular parameters	PPD: 6–10 mm					
		Radiographic parameters	MD					
		Crevicular parameters	MD					
lamin A., 2017 Clin Exp Dent Res. Case-control study [28] Low risk Grant from Research Council of Norway, Oslo, Norway	Population: n.17 Mean age: 15.4 ± 1.6 yo Gender ratio: 8M/9F Country/Ethnicity: African n.4, Afro-Arab n.13 Comorbidities: none n.17 Anamnesis for infectious disease: none n.17 Therapy for infectious disease: NA	Periodontal conditions: periodontitis n.17		N. of sample: n.68 Type of sample: subgingival n.68 Sampling methods: sterile paper points n.68 Time after periodontal treatment: none n.17 Microbiological identification technique: LAMP method n.17 Sampled site: deepest periodontal site n.17 Target: 16S rRNA n.17	Population: n.17 Mean age: 15.6 ± 1.5 yo Gender ratio: 7M/10F Country/Ethnicity: African n.4, Afro-Arab n.13 Comorbidities: none n.17 Anamnesis for infectious disease: none n.17 Therapy for infectious disease: NA	Periodontal conditions Clinically healthy periodontium n.17		N. of sample: n.68 Type of sample: subgingival n.68 Sampling methods: sterile paper points n.68 Time after periodontal treatment: none n.17 Microbiological identification technique: LAMP method n.17 Sampled site: deepest periodontal site n.17 Target: 16S r-RNA n.17
		Periodontal parameters:				Periodontal parameters:		
		Clinical parameters	PPD: 6.9 ± 1.5 mm			Clinical parameters	PPD: 1.4 ± 0.5 mm	
			CAL: −4.8 ± 1.2 mm				BOP: 0	
			BOP (% of positive sites): 62.4%					
		Radiographic parameters	MD			Radiographic parameters	MD	
		Crevicular parameters	MD			Crevicular parameters	MD	

Michalowicz B.S., 2000 J Periodontal Case-control study [24] Moderate risk No funding	Population: n.35 Mean age: 14–18 yo Gender ratio: MD Country/Ethnicity: North-Central Jamaica n.35 Comorbidities: none n.35 Anamnesis for infectious disease: none n.35 Therapy for infectious disease: NA	Periodontal conditions: periodontitis n.35		N. of sample: n.140 Type of sample: subgingival n.140 Sampling methods: sterile paper points n.140 Time after periodontal treatment: none n.35 Microbiological identification technique: nested PCR n.35 Sampled site: deepest periodontitis sites n.35 Target: 16S rRNA, leukotoxin operon n.35	Population: n.65 Mean age: 14–18 yo n.65 Gender ratio: MD Country/Ethnicity: North-Central Jamaica n.65 Comorbidities: none n.65 Anamnesis for infectious diseases: MD Therapy for infectious diseases: MD	Periodontal conditions: Clinically healthy periodontium n.65		N. of sample: n.260 Type of sample: subgingival n.260 Sampling methods: sterile paper points n. 260 Time after periodontal treatment: none n.65 Microbiological identification technique: nested PCR n.65 Sampled site: deepest periodontal sites n.65 Target: 16S rRNA, leukotoxin operon n.65
		Periodontal parameters						
		Clinical parameters	PPD ≥ 5 mm n.35					
						Periodontal parameters		
			CAL ≤ −3 mm n.35			Clinical parameters	PPD ≤ 5 mm n.65	
							CAL ≤ −2 mm n.65	
		Radiographic parameters	MD			Radiographic parameters	MD	
		Crevicular parameters	MD			Crevicular parameters	MD	
	
Otero R. A., 2015 Rev Inst Med Trop Sao Paulo Case-control study [25] Low risk CNPq, CAPES, FAPERJ, Brazil	Population: n.16 Mean age: N/D Gender ratio: N/D Country/ethnicity: MD Comorbidities: None n.16 Anamnesis for infectious disease: none n.16 Therapy for infectious diseases: MD	Periodontal conditions: Gingivitis n.16		N. of sample: n.16 Type of sample: saliva n.16 Sampling methods: sterile container n.16 Time after periodontal treatment: none n.16 Microbiological identification technique: PCR n.16 Sampled site: MD Target: MD	Population: n.32 Mean age: N/D Gender ratio: N/D Country/ethnicity: MD Comorbidities: None n.32 Anamnesis for infectious disease: none n.32 Therapy for infectious diseases: NA	Periodontal conditions: Clinically healthy periodontium n.32		N. of sample: n.32 Type of sample: saliva n.32 Sampling methods: sterile container n.32 Time after periodontal treatment: none n.32 Microbiological identification technique: PCR n.32 Sampled site: MD Target: MD
		Periodontal parameters				Periodontal parameters		
		Clinical parameters	BoP 1 n.16			Clinical parameters	MD	
		Radiographic parameters	MD			Radiographic parameters	MD	
		Crevicular parameters	MD			Crevicular parameters	MD	
Radvar M., 2006 Intl. J, Virol. Case-control study [26] Low risk No funding	Population: n.1 Mean age: 17 yo Gender ratio: 1F Country/Ethnicity: Iran n.1 Comorbidities: none n.1 Anamnesis for infectious disease: none n.1 Therapy for infectious diseases: NA	Periodontal conditions: Periodontitis n.1		N. of sample: n.3 Type of sample: Subgingival n.3 Sampling methods: sterile paper points n.3 Time after periodontal treatment: none n.1 Microbiological analysis technique: nested PCR n.1 Sampled site: periodontal sites with PPD ≥ 6 mm n.1 Target: MD				
		Periodontal parameters:						
		Clinical parameters	PPD ≥ 6 mm n.1					
			BoP 1 n.1					
		Radiographic parameters	MD					
		Crevicular parameters	MD					
Rams T.E., 2024 J Periodontal. Case-control study [27] Low risk No funding	Population: n.22 Mean age: 16 ± 1.5 yo, range 14–18 yo Gender ratio: 11M/11F Country/Ethnicity: Southwestern America (Pueblo Indian population) n.22 Comorbidities: none n.22 Anamnesis for infectious disease: MD Therapy for infectious diseases: MD	Periodontal conditions: MIPP n.22		N. of sample: n.66 Type of sample: Subgingival n.66 Sampling methods: sterile paper points n.66 Time after periodontal treatment: none n.22 Microbiological analysis technique: culture n.22 nested PCR n.8 Sampled site: periodontal sites with PPD ≥ 6 mm and BoP n.22 Target: MD	Population: n.19 Mean age: MD Gender ratio: MD Country/ethnicity: Southwestern America (Pueblo Indian population) n.19 Comorbidities: none n.19 Anamnesis for infectious diseases: MD Therapy for infectious diseases: MD	Periodontal conditions: Clinically healthy periodontium n.19		N. of sample: n.57 Type of sample: Subgingival n.57 Sampling methods: sterile paper points n.57 Time after periodontal treatment: none n.19 Microbiological analysis technique: culture n.19 nested PCR n.4 Sampled site: periodontal sites with PPD ≤ 3.5 mm and BoP 0 n.19 Target: MD
		Periodontal parameters:				Periodontal parameters		
		Clinical parameters	PPD 7.2 ± 0.9 mm			Clinical parameters	PPD ≤ 3.5 mm	
			BoP 1				BoP: 0	
		Radiographic parameters	MD			Radiographic parameters	MD	
		Crevicular parameters	MD			Crevicular parameters	MD	
Ting M., 2000 J Periodont Res Case-control study [17] Serious risk No funding	Population: n.8 Mean age: 13.38, range 10–16 yo Gender ratio: 3M/5F Country/Ethnicity: African-American n.7; Persian n.1 Comorbidities: none n.8 Anamnesis for infectious disease: none n.8 Therapy for infectious diseases: NA	Periodontal conditions: MIPP n.8		N. of sample: n.24 Type of sample: Subgingival n.24 Sampling methods: sterile paper points n.24 Time after periodontal treatment: none n. 8 Microbiological analysis technique: culture n.8 nested PCR n.8 Sampled site: periodontal sites in molars and incisors n.8 Target: CMV DNA, CMV mRNA, mRNA for CMV late major capsid protein gene, EBNA gene, 16 S rRNA n. 8				
		Periodontal parameters:						
		Clinical parameters	PPD 5–9 mm					
		Radiographic parameters	MD					
		Crevicular parameters	MD					


**Abbreviation:** number “n.”, male “M”, female “F”, years old “yo”, missing data “MD”, not applicable “NA”, not defined “N/D” cytomegalovirus “CMV”, major capsid protein “MCP”, immediate early “IE”, reverse transcription “RT”, pocket probing depth “PPD”, bleeding on probing “BoP”, Full Mouth Bleeding Score “FMBS”, Full Mouth Plaque Score “FMPS” Calculus Index Simplified “CI-S”, molar–incisor pattern periodontitis “MIPP”, human immunodeficiency virus “HIV”, highly active antiretroviral therapy “HAART”, loop-mediated isothermal amplification “LAMP”, Dedicator Of Cytokinesis 8 “DOCK 8”.

**Table 2:** Microbiological content of gingivitis/periodontitis and clinically healthy periodontium group sorted by culture (positivity, counts) and PCR (positivity, counts): Study (first author, year, periodontal condition; sample size; microbiological analysis technique); microorganisms (viruses—HHV, coinfections, other viruses; periodontal pathogen bacteria complexes—red, orange, yellow, green, purple and outliers bacteria), total anaerobic bacteria count, total bacteria count.

Study	Microorganisms	Outcome(s)							
		Gingivitis/Periodontitis Group				Clinically Healthy Periodontium Group			
		Culture		PCR		Culture		PCR	
		Positivity	Count	Positivity	Count	Positivity	Count	Positivity	Count
Contreras A., 1998 Oral Microobiol Immunol [23] MIPP Intervention group n.3 Microbiological analysis technique: RT-PCR n.3		**Viruses**							
	**HHV:**								
	CMV DNA (acute infection)	NA	NA	Positive n.3	MD				
	CMV mRNA (latency)	NA	NA	Positive n.2 Negative n.1	MD				
Elamin A., 2017 Clin Exp Dent Res. [28] Periodontitis Intervention group: n.17 Microbiological analysis technique: LAMP method n.17 Control group: n.17 Microbiological analysis technique: LAMP method n.17		**Viruses**							
	HHV:								
	EBV-I	NA	NA	Positive n.11 Negative n.6	MD	NA	NA	Positive n.8 Negative n.9	MD
	CMV	NA	NA	Positive n. 12 Negative n.5	MD	NA	NA	Positive n.2 Negative n.15	MD
	HHV Coinfections:								
	EBV-I + CMV	n.8				MD			
		**Periodontal pathogen bacteria**							
	**Red complex species**								
	*Porphyromonas gingivalis*	None	None	Positive n.14 Negative n.3	MD	None	None	Positive n.7 Negative n.10	MD
	*Tannerella forsythia*	None	None	Positive n.13 Negative n.4	MD	None	None	Positive n. 9 Negative n.8	MD
	*Treponema denticola*	None	None	Positive n.12 Negative n.5	MD	None	None	Positive n.14 Negative n.3	MD
	**Outliers complex species**								
	*Aggregatibacter actinomycetemcomitans*	None	None	Positive n.12 Negative n.5	None	None	None	Positive n.1 Negative n.16	None
Michalowicz B.S., 2000 J Periodontal [24] Periodontitis Intervention group: n.35 Microbiological analysis technique: Nested PCR n.35 Control group: n.65 Microbiological analysis technique: Nested PCR n.65		**Viruses**							
	HHV:								
	CMV	NA	NA	Positive n.19 Negative n.16	MD	NA	NA	Positive n.14 Negative n.51	MD
	EBV-I	NA	NA	Positive n.14 Negative n.21	MD	NA	NA	Positive n.11 Negative n.54	MD
		**Periodontal pathogen bacteria**							
	**Red complex species**								
	*Porphyromonas gingivalis*	None	None	Positive n.27 Negative n.8	MD	None	None	Positive n.22 Negative n.43	MD
	**Outliers bacterial species**								
	*Aggregatibacter actinomycetemcomitans*	None	None	Positive n.18 Negative n.17	MD	None	None	Positive n.13 Negative n.52	MD
Otero R.A., 2015 Rev Inst Med Trop Sao Paulo [25] Gingivitis Intervention group: n.16 Microbiological analysis technique: PCR n.16 Control group n.32 Microbiological analysis technique: PCR n.32		**Viruses**							
	HHV:								
	HSV-I	NA	NA	Positive n.3 Negative n.13	MD	NA	NA	Positive n.32	
	HSV-II	NA	NA	Positive n.3 Negative n.13	MD	NA	NA	Positive n.2 Negative n.30	
	CMV	NA	NA	Negative n.16	MD	NA	NA	Positive n.5 Negative n.27	
	EBV	NA	NA	Negative n. 16	MD	NA	NA	Negative n.32	None
	VZV	NA	NA	Negative n.16	MD	NA	NA	Negative n.32	None
Radvar M., 2006 Intl. J, Virol. [26] Periodontitis Intervention group n.1 Microbiological analysis technique: nested PCR n.1 Control group: n.1 Microbiological analysis technique: nested PCR n.1		**Viruses**							
	HHV:								
	CMV	NA	NA	Positive n. 1	MD				
	EBV-I	NA	NA	Negative n.1	MD				
	HSV	NA	NA	Negative n.1	MD				
Rams T.E., 2024 J Periodontal. [27] MIPP Intervention group: n.22 Microbiological analysis technique culture n.22, nested PCR n.8 Control group: n.19 Microbiological analysis technique: Culture n. 19 Nested PCR n.4		**Viruses**							
	**HHV:**								
	CMV	NA	NA	Positive n.2 Negative n.6	MD	NA	NA	Negative n.4	MD
	EBV-I	NA	NA	Positive n.1 Negative n.7	MD	NA	NA	Negative n.4	MD
	EBV-II	NA	NA	Negative n.8	MD	NA	NA	Negative n.4	MD
	HSV	NA	NA	Negative n.8	MD	NA	NA	Negative n.4	MD
	HHV Coinfections:								
	CMV + EBV-I	n.1				None			
		**Periodontal pathogen bacteria**							
	**Red complex species**								
	*Porphyromonas gingivalis*	Positive n.14 Negative n.8	5.80 ± 7.00 log_10_/mL n.14	MD	MD	Positive n.1 Negative n.18	7.5 (log_10_/mL) n.1	MD	MD
	*Tannerella forsythia*	Positive n.14 Negative n.8	1.8 ± 2.3 log_10_/mL n.14	MD	MD	Negative n.19	0	MD	MD
	**Orange complex species**								
	*Prevotella intermedia/nigriscens*	Positive n.19 Negative n.3	4.3 ± 4.2 log_10_/mL n.19	MD	MD	Positive n.7 Negative n.12	1.0 ± 0.9 (log_10_/mL) n.7	MD	MD
	*Parvimonas micra*	Positive n.16 Negative n.6	7.1 ± 6.9 log_10_/mL n.16	MD	MD	Positive n.4 Negative n.15	3.3 ± 1.7 (log_10_/mL) n.4	MD	MD
	*Fusobacterium nucleaatum*	Positive n.21 Negative n.1	2.6 ± 3.1 log_10_/mL n.21	MD	MD	Positive n.9 Negative n.10	1.4 ± 1.2 (log_10_/mL) n.9	MD	MD
	*Campylobacter rectus*	Positive n.16 Negative n.6	4.8 ± 3.8 log_10_/mL n.16	MD	MD	Positive n.3 Negative n.16	2.2 ± 1.5 (log_10_/mL) n.3	MD	MD
	*Eubacterium species*	Positive n.3 Negative n.19	3.2 ± 1.8 log_10_/mL n.3	MD	MD	Negative n.19	0	MD	MD
	**Green complex species**								
	*Eikenella corrodens*	Positive n.2 Negative n.20	3.0 ± 4.1 log_10_/mL n.2	MD	MD	Negative n.19	0	MD	MD
	**Outliers bacterial species**								
	*Aggregatibacter actinomycetemcomitans*	Positive n.10 Negative n.12	3.1 ± 3.3 log_10_/mL n.10	MD	MD	Positive n.1 Negative n.18	0.1 (log_10_/mL) n.1	MD	MD
	*β-hemolytic streptococci*	Positive n.7 Negative n.15	3.4 ± 5.4 log_10_/mL n.7	MD	MD	Positive n.1 Negative n.18	1.1 (log_10_/mL) n.1	MD	MD
	*Capnocytophaga species*	Positive n.10 Negative n.12	3.0 ± 3.2 log_10_/mL n.10	MD	MD	Positive n.6 Negative n.13	1.2 ± 1.1 (log_10_/mL) n.6	MD	MD
	*Prevotella dentalis*	Positive n.3 Negative n.19	2.5 ± 3.7 log_10_/mL n.3	MD	MD	Negative n.19	0	MD	MD
	*Gram-negative enteric rods*	Positive n.1 Negative n.21	8.3 log_10_/mL n.1	MD	MD	Negative n.19	0	MD	MD
	*Viridans streptococci plus*	Positive n.22	38.8 ± 11.7 log_10_/mL n.22	MD	MD	Positive n.19	77.3 ± 12.7 (log_10_/mL) n.19	MD	MD
	Total anaerobic bacteria count:	NA	7.97 ± 0.55 log_10_/mL n.22	MD	MD	NA	7.49 ± 0.30 (log_10_/mL) n.19	MD	MD
Ting M., 2000 J Periodont Res [17] MIPP Intervention group: n.8 Microbiological analysis technique culture n.8, nested PCR n.8 Control group: n.8 Microbiological analysis technique: Culture n. 8 Nested PCR n.8		**Viruses**							
	**HHV:**								
	CMV DNA	NA	NA	Positive n.7 Negative n.1	MD				
	CMV Mrna (activation)	NA	NA	Positive n.6 Negative n.2	MD				
	EBV-I	NA	NA	Positive n.6 Negative n.2	MD				
	EBV-II	NA	NA	Positive n.1 Negative n.7	MD				
	HSV	NA	NA	Positive n.4 Negative n.4	MD				
	HHV Coinfections:								
	CMV + EBV-1 + HSV	n.2							
	CMV + HSV	n.1							
	CMV + EBV-I + EBV-II	n.2							
	EBV-I + HSV	n.1							
	CMV + EBV-I	n.1							
		**Periodontal pathogen bacteria**							
	**Outliers bacterial species**								
	*Aggregatibacter actinomycetemcomitans*	Positive n.4 Negative n.4	0% * n.4 0.3% * n.1 0.6% * n.1 2.6% * n.1 4.2% n.1	Positive n.6 Negative n.2	MD				

**Abbreviation:** not applicable “NA”, missing data “MD”, MIPP “molar–incisor pattern periodontitis”, human herpesvirus “HHV”, cytomegalovirus “CMV”, herpes simplex virus “HSV”, human papillomavirus “HPV”, varicella zoster virus “VZV”, polymerase chain reaction “PCR”, Epstein–Barr virus “EBV”, percentage “%”, of total counts “*”, millimeters “mL”, logarithm to base 10 “log_10_”, reverse transcription “RT”, pocket probing depth “PPD”, loop-mediated isothermal amplification “LAMP”.

## Data Availability

Data are available in the MEDLINE/PubMed, Scopus, Web of Science, and Cochrane Library.

## Supplementary Materials

The following supporting information can be downloaded at: https://www.mdpi.com/article/10.3390/children12010039/s1, File S1: Quality assessment. Table S1: Quality assessment of included nonrandomized studies according to ROBINS-1. First Author, year, reference, ROBINS-1 bias domain, and quality assessment.
