# Supplementary material for: Human Herpesviruses, Bacteria, and Fungi in Gingivitis and Periodontitis Pediatric Subjects: A Systematic Review
Source: Children (Basel). 2024 Dec 29;12(1):39. doi: 10.3390/children12010039 (PMC11763593; doi:10.3390/children12010039)
Supplement: Supplementary file 1 [file children-12-00039-s001.zip › children-3357424-supplementary.pdf]

Human Herpes Virus, Bacteria and Fungi in gingivitis and periodontitis pediatric subjects: a Systematic Review

Children

Federica Di Spirito [1,*], Massimo Pisano [1†], Mario Caggiano,[1,†] Giuseppina De Benedetto [1], Maria Pia Di Palo [1], Gianluigi Franci [1,*,‡], Massimo Amato [1,‡]

[1] Department of Medicine, Surgery and Dentistry, University of Salerno, Via S. Allende, 84081 Baronissi, SA, Italy; pisano.studio@virgilio.it (M.P.); macaggiano@unisa.it (M.C.); giusydb15@gmail.com (G.D.B.); mariapia140497@gmail.com (M.P.D.P.); mamato@unisa.it (M.A.)

* Correspondence: fdispirito@unisa.it (F.D.S.); gfranci@unisa.it (G.F.)

† These authors contributed equally to this work.
‡ These authors contributed equally to this work.

Supplementary File S1- Quality assessment

Quality assessment of included nonrandomized studies of interventions (Table S1), using the Risk of Bias in Nonrandomized Studies of Interventions (ROBINS-1), and of case reports (Table S2), using the Johanna Briggs Institute (JBI) for case reports.

Table S1. Quality assessment of included nonrandomized studies according to ROBINS-1. First Author, year, reference, ROBINS-1 bias domain, and quality assessment.

	Conteras A., 1998 [23]	Elamin A., 2017 [28]	Michalowicz B.S., 2000 [24]	Otero R.A, 2015 [25]	Radvar M., 2006 [26]	Rams T.E., 2024 [27]	Ting M., 2000 [17]
**Domain 1**							
1.1	N	N	N	N	N	N	N
1.2	NA	NA	NA	NA	NA	NA	NA
1.3	NA	NA	NA	NA	NA	NA	NA
1.4	NA	NA	NA	NA	NA	NA	NA
1.5	NA	NA	NA	NA	NA	NA	NA
1.6	NA	NA	NA	NA	NA	NA	NA
1.7	NA	NA	NA	NA	NA	NA	NA
1.8	NA	NA	NA	NA	NA	NA	NA
Domain 1 Judgment	Low risk	Low risk	Low risk	Low risk	Low risk	Low risk	Low risk
**Domain 2**							
2.1	N	N	N	N	N	N	N
2.2	NA	NA	NA	NA	NA	NA	NA
2.3	NA	NA	NA	NA	NA	NA	NA
2.4	NI	PY	NI	Y	Y	NI	NI
2.5	NA	NA	NA	NA	NA	NA	NA
Domain 2 Judgment	Moderate risk	Low risk	Moderate risk	Low risk	Low risk	Moderate risk	Moderate risk
**Domain 3**							
3.1	PY	Y	Y	Y	Y	Y	PN
3.2	PY	Y	Y	Y	Y	Y	Y
3.3	N	N	N	N	N	N	N
Domain 3 Judgment	Low risk	Low risk	Low risk	Low risk	Low risk	Low risk	Serious risk
**Domain 4**							
4.1	N	N	N	N	N	N	N
4.2	NA	NA	NA	NA	NA	NA	NA
4.3	NA	NA	NA	NA	NA	NA	NA
4.4	NA	NA	NA	NA	NA	NA	NA

4.5	NA	NA	NA	NA	NA	NA	NA
4.6	NA	NA	NA	NA	NA	NA	NA
Domain 4 Judgment	Low risk	Low risk	Low risk	Low risk	Low risk	Low risk	Low risk
**Domain 5**							
5.1	Y	Y	Y	Y	Y	Y	Y
5.2	N	N	N	N	N	N	N
5.3	N	N	N	N	N	N	N
5.4	NA	NA	NA	NA	NA	NA	NA
5.5	NA	NA	NA	NA	NA	NA	NA
Domain 5 Judgment	Low risk	Low risk	Low risk	Low risk	Low risk	Low risk	Low risk
**Domain 6**							
6.1	N	N	N	N	N	N	N
6.2	Y	Y	Y	Y	Y	Y	Y
6.3	Y	Y	Y	Y	Y	Y	Y
6.4	N	N	N	N	N	N	N
Domain 6 Judgment	Low risk	Low risk	Low risk	Low risk	Low risk	Low risk	Low risk
**Domain 7**							
7.1	N	N	N	N	N	PN	N
7.2	PN	N	N	N	PN	PN	N
7.3	N	N	N	N	N	N	N
Domain 7 Judgment	Low risk	Low risk	Low risk	Low risk	Low risk	Low risk	Low risk
**Quality assessment**	Moderate risk of bias	Low risk of bias	Moderate risk of bias	Low risk	Low risk of bias	Low risk of bias	Serious risk of bias

Abbreviations: yes “Y”; no “N”; Not Applicable “NA”; Probably yes “PY”; Probably no “PN”; No Information “NI”
